# Impact of Low-Level Ergot Alkaloids and Endophyte Presence in Tall Fescue Grass on the Metabolome and Microbiome of Fall-Grazing Steers

**DOI:** 10.3390/toxins17050251

**Published:** 2025-05-17

**Authors:** Ignacio M. Llada, Jeferson M. Lourenco, Madison M. Dycus, Jessica M. Carpenter, Zachery R. Jarrell, Dean P. Jones, Garret Suen, Nicholas S. Hill, Nikolay M. Filipov

**Affiliations:** 1 Interdisciplinary Toxicology Program, University of Georgia, Athens, GA 30602, USA; ignacio.llada@uga.edu; 2 Department of Physiology and Pharmacology, University of Georgia, Athens, GA 30602, USA; jmc69554@uga.edu; 3 Department of Animal and Dairy Science, University of Georgia, Athens, GA 30602, USA; jefao@uga.edu (J.M.L.); madison.dycus@uga.edu (M.M.D.); 4 Department of Medicine, Division of Pulmonary, Allergy, Critical Care & Sleep Medicine, Emory University, Atlanta, GA 30322, USAdpjones@emory.edu (D.P.J.); 5 Department of Bacteriology, University of Wisconsin-Madison, Madison, WI 53706, USA; gsuen@wisc.edu; 6 Department of Crop and Soil Sciences, College of Agriculture, University of Georgia, Athens, GA 30602, USA; nhill@uga.edu

**Keywords:** tall fescue, *Epichloë coenophiala*, fescue toxicosis, metabolomics, microbiome

## Abstract

Fescue toxicosis (FT) is a mycotoxin-related disease caused by the ingestion of tall fescue, naturally infected with the ergot alkaloid (EA)-producing endophyte *Epichloë coenophiala*. Some grazing on endophyte-free (E−) or non-toxic (NT), commercial endophyte-infected pastures takes place in the US as well. Earlier, we found that grazing on toxic fescue with low levels of EAs during fall affects thermoregulation, behavior, and weight gain. Building on these findings, the current study aimed to investigate how the presence of low EA-producing E+ or NT endophytes can influence animal metabolome, microbiome, and, ultimately, overall animal health. Eighteen Angus steers were placed on NT, E+, and E− fescue pastures for 28 days. Urine, rumen fluid (RF), rumen solid (RS), and feces were collected pre-exposure, and on days 2, 7, 14, 21, and 28. An untargeted high-resolution metabolomics approach was used to analyze urine and RF, while 16S rRNA-based next-generation sequencing (NGS) was used to examine RF, RS, feces, and fescue plant microbiomes. While alpha- or beta-microbiota diversity across all analyzed matrices were unaffected, there were specific effects of E+ on the relative abundance of some taxa (i.e., *Prevotellaceae*). Additionally, E+ grazing impacted aromatic amino acid metabolism in the urine and the metabolism of lipids in both the RF and urine. In both matrices, trace amine-related metabolic features differed markedly between E+ and the other groups. Compared to the endophyte-free group, endophyte presence, whether novel or toxic, influenced amino acid and carbohydrate metabolism, as well as unsaturated fatty acid biosynthesis. These findings suggest that low-EA-producing and non-toxic endophytes in fescue have more prominent effects on the metabolome than the microbiome, and this metabolome perturbation might be associated with decreased performance and reported physiological signs of FT.

## 1. Introduction

Fescue toxicosis (FT) is a mycotoxin-related disease caused by the ingestion of tall fescue plants infected with the ergot alkaloid (EAs)-producing endophyte *Epichloë coenophiala* [1], which dominates fescue pastures in the US and other parts of the world. The plant and fungus coexist symbiotically: the host provides nutrients and a means of dissemination, while the fungus, through the production of secondary metabolites, offers protection to the plant against both biotic and abiotic stressors [2]. However, some of the endophyte-derived metabolites also have adverse effects on animal health and, consequently, on their productivity [3]. EAs are the main toxins associated with this pasture-related pathology [4]. Although ergovaline (EV) is considered the primary EA involved, other alkaloids [5] may also contribute. Since EV constitutes 10% to 50% of the total EA concentration in the plant [6], and its toxic dietary levels are proposed to be between 400 and 750 ppb [7], little or no toxicity should be observed when total EA concentrations are markedly lower. However, no specific threshold levels for total EA have been established. The most well-known mechanism of action of EAs is their ability to bind to and mimic the actions of biogenic amines, such as serotonin, (nor) epinephrine, and dopamine at their receptors [3]. These interactions lead to, among others, peripheral and core vasoconstriction [8,9], disrupted thermoregulation [10], altered nutrient absorption [11], reduced intake [12], and impaired behavior [13]. Despite the research progress, the pathophysiology of FT remains incompletely understood.

The use of high-resolution metabolomics (HRM) has emerged as a promising approach in the investigation of the pathogenesis of this disease. This methodology allows the exploration of a broad spectrum of metabolites in biological samples without predefined targets, enabling the identification of biomarkers and clarification of disrupted metabolic pathways. Recent studies, including ours, revealed alterations in lipid and amino acid metabolism, especially tyrosine and tryptophan, in steers grazing toxic fescue [14,15,16]. These in vivo studies were conducted on pastures with total EAs exceeding 1000 ppb; however, the effects of low EA levels on metabolome remain known.

When considering ruminants, it is vital to consider their symbiotic relationship with a diverse community of microorganisms (MOs) in their forestomach and hindgut. These MOs play a key role in the breakdown of plant polysaccharides [17], releasing volatile fatty acids (VFAs) that serve as the primary energy source [18]. Thus, MOs’ presence and function are critical for ruminant’s health and productivity. Despite their significance, MOs’ susceptibility to natural toxins, like EAs, is underexplored. Although studies report disruption in the rumen [16,19] and fecal [15] microbiome in cattle grazing high-EA fescue, the impact of low EA levels on the beef cattle microbiome has not been investigated.

Management strategies to mitigate the effects of this disease have been explored [20], including the use of a non-toxic (NT) endophyte that produces fewer or no measurable EAs. This novel fungus was introduced in a fescue grass and is available on the market as new cultivars. Studies have shown that NT effectively mitigates the negative effects of E+ on grazing animals and maintains the desired performance for farmers, especially weight gain [21,22]. Like the wild-type endophyte, NT can still produce plant-protective compounds, like peramine, preserving the persistence and agronomic qualities of the plant [21]. Despite its benefits, there is no information on the effects of introducing a novel organism that produces plant-protective alkaloids and other secondary metabolites into the rumen, particularly regarding its impact on the animal’s microbiome and metabolome.

Recently [23], as a part of this trial, we reported that Angus steers grazing E+ with low levels of EAs gained on average 60% less than those on NT and E−. E+ steers exhibited behavioral deficits indicative of less-than-optimal grazing patterns, higher rectal temperature, and lower skin surface temperature over a 28-day study [23]. Notably, NT steers, while maintaining weight gain similar to the control (E−), had an apparent better thermoregulation result than either of the other two (E+ or E−) groups [23]. Employing high-resolution metabolomics (HRM) in the rumen fluid and urine, and next-generation sequencing (NGS) in the rumen content and feces of the same steers, our objectives were to investigate the impact of toxic, low-ergot-alkaloid (EA)-producing, and non-toxic endophytes on the grazing steers’ metabolome and microbiome. We also explored the associations between microbiome and metabolomics features with the physiological parameters affected by E+ grazing reported in [23]. This study will help determine if low EA ingestion and endophyte presence, whether novel or toxic, impact the metabolome and/or microbiome in a way that will affect the health and performance of tall fescue-grazing steers.

## 2. Results

### 2.1. Physiological Parameters

Both cumulative and average daily weight gain (ADG) were significantly lower (*p* ≤ 0.01) in steers grazing E+ (>60% decrease) than in steers grazing NT or E− fescue. E+ steers also had higher rectal temperatures than E− and NT, and lower skin surface temperatures than NT post-pasture placement. For more details about the E+ fescue-grazing impact on these physiological parameters, refer to [23].

### 2.2. Endophyte and Total Plant Ergot Alkaloids

The mean endophyte presence in the E+ pastures was 78%, and all endophyte-infected tillers tested positive for EA. Mean endophyte presence in the NT pastures was 92% with no EA production, and the E− paddocks had 0% endophyte presence. The total EA concentration in the whole tissue plant samples was 12.6 parts per billion (ppb) for E+, while for NT and E− pastures, the levels were below the detection limit for the test.

### 2.3. Metabolomics Results

**Figure 1:**
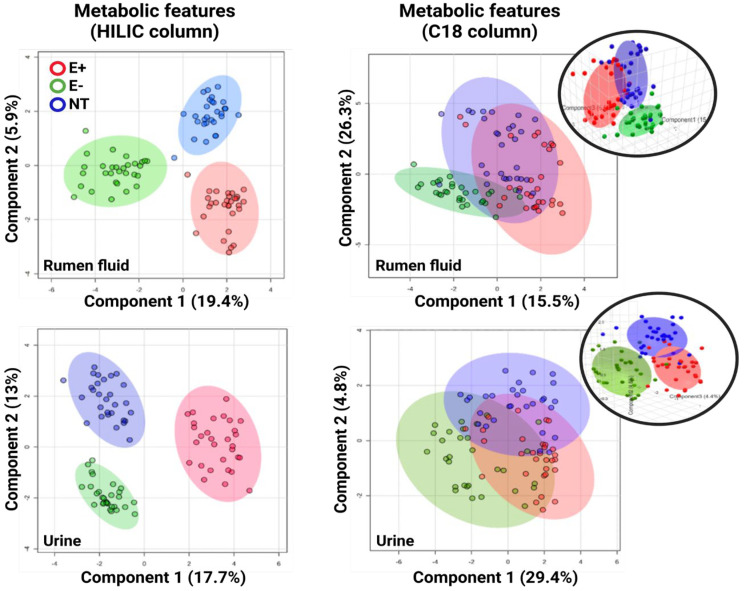
sPLS-DA (sparse partial least squares discriminant analysis) plots using the metabolic features from: (**left**) the HILIC column and (**right**) C18 column for the ruminal fluid and urine of steers grazing toxic endophyte-infected tall fescue (E+; n *=* 6), non-toxic endophyte-infected tall fescue (NT; n = 6), and endophyte-free tall fescue (E−; n = 6). Additionally, a 3D plot for each matrix is presented for the C18 column (insets).

After HRM processing, 12,094 (HILIC) and 5348 (C18) unique features (i.e., metabolites with unique *m/z* and retention times) were detected in the urine. Of these, 114 (HILIC) and 118 (C18) features were significantly different between groups (*p*-value (FDR) cutoff: 0.05). In the rumen fluid, 13,821 (HILIC) and 7221 (C18) unique features were detected, with 147 (HILIC) and 61 (C18) of them being significantly different between treatments. Overall, sPLS-DA analysis showed a clear group separation in both urine and rumen samples after using the metabolic features obtained from the HILIC column. After using features from the C18 column in both matrices, an overlap was observed between the E+ and NT groups; the E− group remained distinct (Figure 1). On days 2, 7, 14, 21, and 28 post-pasture placements, this consistent pattern persisted across groups, while for both columns, a clear group overlap pre-pasture placement was noted.

**Figure 2:**
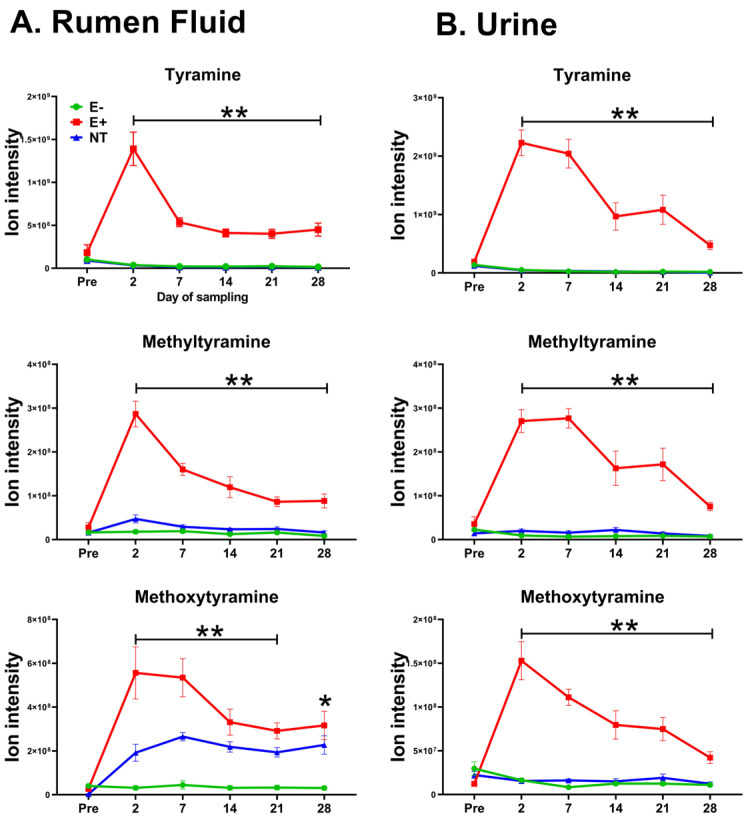
Trace amines identified by an untargeted metabolomic approach in (**A**) the rumen fluid and (**B**) urine of steers (n = 18) grazing toxic endophyte (E+; n = 6), non-toxic endophyte (NT; n = 6), and endophyte-free tall fescue (E−; n = 6). (*) indicates a significant difference (*p* ≤ 0.05) between the E+ group and one of the other groups, while (**) indicates a significant difference (*p* ≤ 0.05) between the E+ group and the rest. Data are presented as mean ± SEM.

After multiple comparisons between groups (FDR-corrected *p*-value cutoff: 0.05), 13 rumen fluid and 23 urine metabolic features were most influenced by E+ toxin(s). These features were not different before pasture placement (*p >* 0.05); however, significant differences emerged after they started grazing E+. Table 1 summarizes the metabolic features and their corresponding annotations in rumen fluid and urine that (a) exhibited discriminatory power in the sPLSDA analysis, (b) were influenced by the effect of E+ toxin(s), and (c) showed differences (higher means) post- but not pre-pasture placement in E+ grazing animals. Of these, five overlapped between rumen fluid and urine when considering their *m/z* values (112.0757, 155.1179, 169.1335, 185.1285, 197.1285). Some overlapping metabolic features are presented in Figure 2, while extracted ion chromatograms of the metabolic features from rumen fluid and urine listed in Table 1 are shown in Figure S1 and Figure S2 (Supplementary File S1).

Within E+ steers, two rumen fluid metabolic features were negatively correlated (*r >* −0.4; *p <* 0.05), and one was positively correlated (*r =* 0.4; *p <* 0.05) with the rectal temperature. In urine, only two features were negatively correlated with weight gain (*r >* −0.45; *p* < 0.05). The metabolic features associated with these correlations are listed in Table 2.

Within NT steers, 50 rumen fluid and 20 urine metabolic features were associated with this group, as they only showed statistical significance after comparison with the negative control (E−). Three rumen (*r ≥* 0.55, *p <* 0.05) and one urine (*r =* 0.4, *p <* 0.05) metabolites were significantly positively correlated with skin surface temperature. The rumen metabolites include *m/z* 480.3445, 36 s (Lysophosphatidylcholine, HMDB10407), *m/z* 314.2336, 171 s (Decanoylcarnitine, HMDB00651), and *m/z* 328.1951, 204 s (Ornithine, LMDB00099). The urine metabolite (*r >* 0.4; *p <* 0.05) was *m/z* 169.0586, and 78.5 s (L-Glutamine, LMDB00202). Representative extracted ion chromatograms from metabolic features associated with skin surface temperature are presented in Figure S3 (Supplementary File S1).

**Figure 3:**
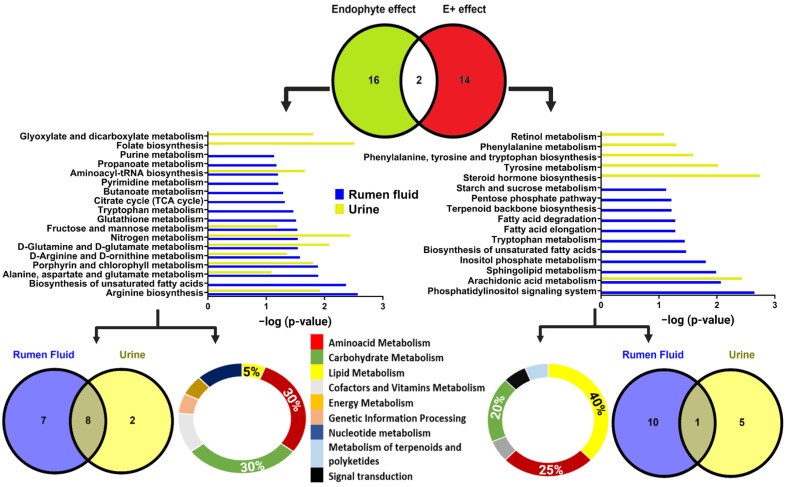
Metabolic pathway influenced by the presence of the endophyte (**left panel**) and the effects of E+ toxin(s) (**right panel**) on the ruminal fluid (blue bars) and urine (yellow bars) of steers. The bottom panel includes, for each bar plot, a Venn diagram, showing the number of pathways affected within each matrix, and the ones that overlapped, and a pie chart showing the proportions (%) by which these pathways were impacted. The negative log of the FDR-corrected *p*-value for each metabolic pathway is on the x-axis of the bar plot graphs.

Regarding pathway analysis, 18 were affected by endophyte presence (E+ or NT), with 8 in rumen fluid and 10 in urine. Eight pathways overlapped between urine and rumen fluid (Figure 3). Of the total affected pathways, 30% were related to amino acid, 30% to carbohydrates, 5% to lipid metabolism, and the rest in other classes. Due to E+ toxin(s), 16 pathways were significantly impacted: 11 in rumen fluid, 6 in urine, and 1 overlapping. Of these, 40% were related to lipids, 25% to amino acids, 20% to carbohydrate metabolism, and the rest in other pathways. The mean levels of metabolites within each pathway influenced by E+ toxin(s) were analyzed to determine whether they were up- or downregulated. Most pathways were downregulated in response to E+ exposure, except for tryptophan metabolism in the rumen fluid, which was upregulated. Biosynthesis of unsaturated fatty acids and tryptophan overlapped between both (E+ and NT) groups (Figure 3).

Pathways related to carbohydrate metabolism differed between both groups. E+ toxin(s) mainly affected the pentose phosphate pathway, inositol phosphate, and starch and sucrose metabolism. A similar pattern was seen with amino acid metabolism, where tryptophan was the only common pathway between groups. However, tyrosine and phenylalanine metabolism, and the biosynthesis of tyrosine, phenylalanine, and tryptophan were impacted only by E+ toxin(s). E+ toxin(s) also had the greatest impact on lipid metabolism, significantly affecting arachidonic acid metabolism, sphingolipid metabolism, fatty acid elongation, fatty acid degradation, and steroid hormone biosynthesis (Figure 3).

### 2.4. Microbiome Results

#### 2.4.1. 16S rRNA Gene Sequencing

We generated 9,432,051 raw sequences, which resulted in 27,674 high-quality sequences after filtering. The average number of paired sequences per sample was 29,754 (range: 3873, 369,743).

#### 2.4.2. Alpha Diversity

No significant differences (*p* ≥ 0.25) were observed among groups in terms of richness and diversity metrics in any of the analyzed matrices. A summary of the microbial richness and diversity metrics is presented in Table S1 (Supplementary File S2).

#### 2.4.3. Beta Diversity

Overall, the analysis of the Unweighted UniFrac distance matrices showed no significant differences (*p* ≥ 0.15) between treatment in rumen fluid, rumen solid samples, and fescue plants. This indicates that, in these sample types, the community composition was not altered by treatments. For the fecal samples, a significant overall effect was observed (*p* ≤ 0.03), with subsequent pairwise comparisons indicating a trend (*p* ≥ 0.08) toward lower diversity in the E+ group compared to E− and NT groups (Figure S4A–C; Supplementary File S2). There was no treatment by day interaction (*p* ≥ 0.25) in any of the analyzed matrices. The Venn diagram revealed an overlap among treatments in the rumen fluid, solid, and feces microbiota.

#### 2.4.4. Specific Microbial Taxa

In tall fescue plants, no differences (*p* ≥ 0.18) were detected in the relative abundance (RA) of microbial communities at both the family and genus levels.

In the rumen fluid, *Prevotellacea* was the only family whose RA was significantly higher (*p* ≤ 0.04) in the E+ compared to the other groups. At the genus level, the RA levels of *Christensenellaceae_R-7_group* and *Butyrivibrio* were reduced (*p* ≤ 0.002), while *Solibacillus*’s RA increased in E+ steers. Among Archaea, *Methanobrevibacter* was the main taxon, with significantly (*p* ≤ 0.003) and numerically lower RA levels in the E+ group compared to the E− or NT groups, respectively. The second most-predominant taxon was *Methanomethylophilaceae_uncultured*, which was significantly (*p* ≤ 0.02) and numerically higher in the E+ group compared to E− or NT steers, respectively. No significant day effects (*p* ≥ 0.2) were observed for any bacterial or Archaeal taxon, though the genus *Butyrivibrio* showed a numerical reduction on each sampling day in the E+ group (Figure S5B; Supplementary File S2).

In the rumen solid, the *Eubacterium_coprostanoligenes_group* genus showed a significant overall (*p* ≤ 0.007), but not by day (*p* ≥ 0.1), reduction in RA. No differences in Archaea RA were observed (*p* ≥ 0.2).

**Figure 4:**
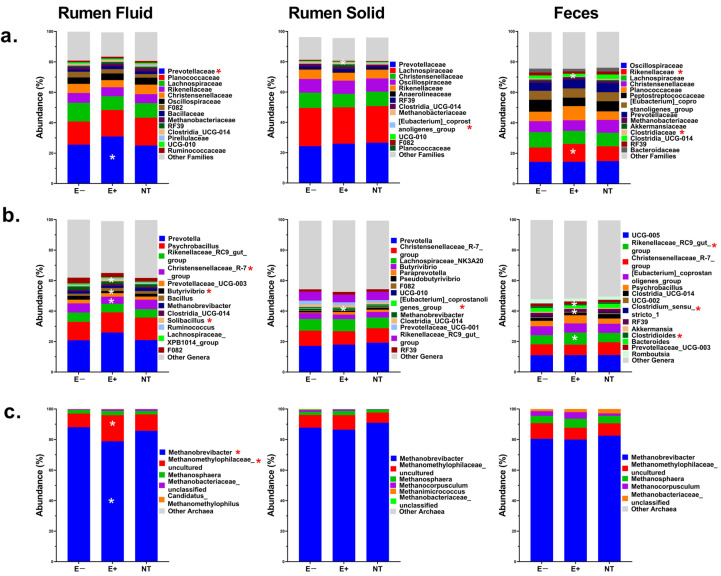
Relative abundance (%) of the main bacterial families (**a**), genera (**b**), and Archaea (**c**) in the rumen fluid, rumen solid, and feces of steers grazing on toxic endophyte (E+; n *=* 6), non-toxic endophyte (NT; n *=* 6), and endophyte-free (E−; n *=* 6) tall fescue. Asterisks (*) indicate significant differences (*p* ≤ 0.05) in the microbial abundance of specific taxa.

In fecal samples, *Rikenellaceae and Clostridiaceae* showed significant changes in their RA levels. *Rikenellaceae* increased (*p* = 0.008), while *Clostridiaceae* decreased (*p* = 0.05) in the E+ group compared to the others. At the genus level, the overall RA of *Rikenellaceae_RC9_gut_group* increased (*p* ≤ 0.01), while *Clostridioides* (*p* ≤ 0.002) and *Clostridium_sensu_stricto_1* were lower (*p* ≤ 0.05) in E+ compared to the other groups (*p* ≤ 0.04). By day of sampling, these differences were most notable on days 14 and 21 (*p* ≤ 0.04). No difference in Archaeal RA was observed. The overall RA of bacterial families and genera, and archaeal genera in rumen fluid, solid, and feces is presented in Figure 4 and Table S2 (Supplementary File S2). Additionally, the treatment-by-day interaction for some of the taxa described is illustrated in Figure S5A,B (Supplementary File S2).

None of the bacteria and archaea that showed significant differences were correlated with physiological parameters (*r* ≤ 0.3, *p* ≥ 0.1). However, in rumen fluid, *Prevotellaceae*, *Methanobrevibacter*, and *Methanomethylophilaceae_uncultured*, and the *Eubacterium_coprostanoligenes_group* in the rumen solid, were positively or negatively correlated (*r* > +/−0.3, *p* ≤ 0.05) with the rumen metabolic features that exhibited greater discrimination due to E+ toxin(s). In feces, *Rikenellaceae_RC9_gut_group* and *Clostridium_sensu_stricto_1* were negatively correlated (*r ≥* −0.3, *p* ≤ 0.05) with urine metabolites that exhibited higher discrimination due to E+ toxin(s). For further details, refer to Table 2. 

## 3. Discussion

Overall, the findings from this study reveal that grazing E+ tall fescue with low levels of EAs has a greater impact on the metabolome than the microbiome. While the microbial communities remained stable at a macro-level, the metabolome underwent noticeable changes, suggesting that even low-level alkaloid exposure can disrupt metabolic processes possibly altering animal health and performance. Only the E+ endophyte strain, potentially linked to EA production, affected lipid metabolism and the biosynthesis of three essential aromatic amino acids: phenylalanine, tyrosine, and tryptophan. Additionally, levels of trace amine-related metabolic features differed markedly in the E+ compared to the other two groups. Interestingly, endophyte presence in the fescue grass, whether novel or toxic, influenced animal amino acid levels, carbohydrate metabolism, and the biosynthesis of unsaturated fatty acids. This, along with the overlapping features (C18) between E+ and NT groups highlights the effects of the endophyte, possibly due to the production of non-ergot secondary metabolites, on the metabolome of grazing animals. Although this did not negatively affect weight gain, i.e., in the NT steers, it still affected the animal’s metabolome, and its potential implications, positive or negative, warrant further in-depth investigation.

Lipids are the most energy-dense nutrient for cattle, stored in adipose tissue and mobilized during energy deficits [24,25]. In this study, only the E+ endophyte strain, potentially linked to EA production, affected lipid metabolic pathways. Metabolites in these pathways were mostly downregulated, suggesting a reduction in lipid metabolic activity. While previous studies also noted a disruption in lipid metabolism after grazing E+ [14,26,27], the underlying mechanism remains unclear. A recent study on steers consuming E+ seeds suggests that hormone imbalances, such as insulin dysregulation, may disrupt lipid metabolism, impairing fat mobilization and energy utilization [28]. Adrenergic receptors regulate lipolysis: in human adipose tissue, β1-, β2-, and β3-receptors stimulate it, while the α2-receptor inhibits it [29,30]. Previous studies on cows [31] and rats [32,33] suggest an agonist effect of EAs on α2-adrenergic receptors. While these studies primarily focused on changes in heart rate and blood pressure, α2-adrenergic receptors were also present in adipose tissue, and their agonism could alter fat mobilization. Another explanation is not just decreased mobilization, but also the reduced synthesis of long-chain fatty acids. In mammals, fatty acid synthesis is reduced by fasting [34,35,36], and grazing toxic fescue lowers voluntary feed intake [37,38], possibly mimicking the fasting state in animals. In this sense, Mote et al. [14] observed elevated blood levels of glycerophospholipids in E+ steers, suggesting impaired glycerol utilization by the adipose tissue. Similarly, McLean et al. [26] reported a reduced expression of genes related to fatty acid and cholesterol synthesis in the mesenteric adipose tissue of steers injected with bromocriptine, a synthetic EA. In short, even low EA levels impair lipid metabolism, consistent with the studies showing similar effects at higher EA levels, highlighting their impact regardless of their concentration.

Grazing E+ with low EA levels had no substantial effect on the ruminal microbiome macrostructure, though some bacterial families/genera were impacted, but their correlations with the animals’ physiological parameters recorded [23] were not significant. The family *Prevotellaceae* was more abundant in E+ steers compared to those on non-toxic pastures, as reported in a previous grazing study [16]. An in vitro study investigating ergovaline (EV) degradation using ruminal content identified specific bacteria, including *Prevotella*, which, though less efficient, showed some capacity to degrade EV [39]. This family is among the most abundant in the rumen [40], and though not obligate amino acid fermenters, many can produce enzymes for the catabolism of proteins, peptides, and amino acids [41]. Further research and the close monitoring of the *Prevotellaceae* family following E+ grazing are needed to understand its role in EA degradation. *Methanobrevibacter*, a key methane producer in the rumen, was lower in E+ steers. Although methane is a net energy loss and contributes to greenhouse gases [42], one study found no difference in emissions between E+ and E− animals [43], though, indirect evidence from ruminal VFAs’ concentration suggests an increased methane output after grazing E+ [44]. In contrast, *Methanomethylophilaceae_uncultured* was higher in E+ steers. This genus uses methyl compounds [45,46,47] for methane production, suggesting that EA exposure may shift the ruminal archaeal community toward organisms that rely on alternative methanogenesis pathways, possibly modifying methane emissions and energy efficiency in cattle. More research on EAs’ effect on archaeal populations could benefit sustainable beef production.

Rapid changes in the fecal microbiome of E+ steers were reported after grazing on E+ [15,48]. In our study, possibly due to the low EA levels, only a trend toward reduced beta diversity was observed, suggesting a potential shift in microbial community structure. Interestingly, in E+ steers, a genus that showed a significant reduction in their abundance was *Clostridium_sensu_stricto_1*. While this genus did not correlate with any physiological parameters recorded, it was negatively associated with two urine metabolites that were elevated in E+ steers and negatively correlated with weight gain. The metabolites were 3,4-dimethoxyphenethylamine, an analog of dopamine (DA) [49,50], and salsolinol, a chemical compound derived from DA [51]. A previous study [16] identified L-dopachrome as a central metabolite in the urine of steers grazing on E+ pastures with higher EA levels. While the metabolites identified here are not identical to L-dopachrome, they belong to the dopaminergic pathways, suggesting that even low EA ingestion may influence the metabolism of DA and its derivatives. The evidence shows that EAs stimulate D2 dopamine receptors, contributing to their toxic effects [52,53], and the administration of DA antagonist reverses the effect, enhancing body weight gain and increasing grazing time [54]. Thus, the observed association between *Clostridium sensu stricto 1* and urine metabolites may imply that: (a) even low EA levels may stimulate dopaminergic receptors, altering dopamine-derived metabolites, which may impact certain fecal genera, or (b) low EA levels may directly reduce the abundance of *Clostridium sensu stricto 1*, a genus that could be involved in the metabolism of these dopamine metabolites.

Metabolic pathways related to plant energy extraction, such as starch and sucrose metabolism and the pentose phosphate pathway, were altered in the rumen of E+ steers. Carbohydrates, including starch and structural fibers, make up over 70% of ruminant diet and are the primary energy source. Microorganisms in the rumen and hindgut break down plant fiber into hexoses and pentoses [17], which are fermented to produce volatile fatty acids (VFAs), the main energy source for maintenance and production. Hexoses are metabolized to pyruvate via glycolysis [55], producing various VFAs [17,18], while pentoses are processed through the pentose phosphate pathway, primarily producing acetate [56]. Thus, disruptions in carbohydrate metabolism can impair the microbes’ ability to extract energy from plant material, disrupting the energy balance within the rumen. This can affect bacterial activity, leading to changes in fermentation pathways and a reduction in microbial efficiency. Lipid metabolic pathways, such as the biosynthesis of unsaturated fatty acids, fatty acid elongation, and degradation, were downregulated in the rumen of E+ steers. The challenge of harvesting energy from plant material may explain this, as it can limit energy availability for microbial growth and activity, including those involved in lipid metabolism and fatty acid biohydrogenation, such as *Butyrivibrio* [24,57], which was significantly reduced in E+ steers. Overall, ingesting low EA levels alters rumen microbial community functionality, resulting in shifts in the rumen metabolome.

Pathways related to the metabolism of arachidonic acid and the biosynthesis of essential aromatic amino acids were affected in the urine of E+ steers. The detection of metabolites from these pathways in the urine of affected animals indicates altered metabolic processes. Several of them were downregulated, suggesting the suppression of their normal activity. The same findings were also observed in other studies with total EAs in pastures > 1000 ppb [14,15,16]. Metabolites associated with arachidonic acid metabolism, such thromboxane A2 (TXA2) [3] and hydroxyeicosatetraenoic acids (HETEs) [14], have already been documented following EA exposure. Our findings emphasize that the dysregulation of arachidonic acid metabolism is possible even in animals grazing pastures with low EA levels, but whether this dysregulation is induced by inflammation-mediated feedback inhibition [58], oxidative stress-induced suppression of COX/LOX enzymes [59,60], or direct interference with PLA2 activity [61], as observed with other fungal metabolites [62], remains unclear at this time. The biosynthesis of phenylalanine, tyrosine, and tryptophan tended to decrease in our study. These amino acids are essential for protein synthesis and serve as precursors for important biomolecules, hormones, and neurotransmitters, including biogenic amines (BAs) like epinephrine, norepinephrine, dopamine, and serotonin [63]. Structural similarity between EAs and BAs allows EAs to bind BAs receptors, potentially signaling excess BAs and triggering feedback that reduces the demand for these amino acids in the synthesis of key molecules. On the other hand, in the rumen fluid of E+ steers, tryptophan metabolism was upregulated. Metabolomic studies have already shown disruption in tryptophan metabolism in steers grazing toxic fescue [14]. One potential explanation might be a microbial breakdown of EV or other alkaloids (e.g., Clavines). Since these compounds are biosynthesized from L-tryptophan during ergoline ring formation, microbial degradation might interfere with this process, releasing tryptophan-derived fragments into the rumen. Indeed, Harlow et al. [39] found that adding EV dramatically enriches tryptophan-utilizing bacteria (by ≈10 fold). This selective shift in the rumen microbiome might increase tryptophan catabolism and provide new tryptophan or indole precursors from alkaloids degradation in the rumen.

In steers grazing E+, five metabolites were identified in both the rumen fluid and urine. Their presence in both biological matrices suggests their potential as future biomarkers. However, to determine their reliability and effectiveness as a diagnostic/prognostic tool, these findings need to be validated in larger studies. The overlapped metabolites tyramine, methyltyramine, methoxytyramine, salsolinol, and 3,4-dimethoxyphenylethylamine (DMPEA) are derivatives of tyrosine or dopamine [49,50,51], suggesting that low EA exposure may alter the metabolism of these neuromodulators. Tyramine, methyltyramine, and methoxytyramine are trace amines (TAs). Similar findings were reported in the plasma and urine of steers grazing toxic fescue in another study [14]. TAs, also referred to as “false transmitters”, are structurally similar to classic vertebrate biogenic amines [64], much like EAs. They primarily function as indirect sympathomimetics by triggering norepinephrine release from nerve terminals, enhancing adrenergic stimulation [65]. TAs also bind to trace amine-associated receptors (TAARs), particularly TAAR1, a G protein-coupled receptors located in the cardiovascular system, central nervous system, and gastrointestinal tract [66]. Interestingly, lysergic acid, the backbone of EAs, has been shown to activate TAAR1 [67]. Regardless of the mechanism, TAs have similar vascular effects to those observed with EAs ingestion. TAs induce blood vessel contractions in many vascular beds, including forearm vessels [68], the aorta [65,69], pig coronary arteries [70], and mesenteric arteries in rats, canines [71,72], and pig [73], and equine digital blood vessels [74]. Beyond vascular effects, tyramine plays a role in gastrointestinal physiology by modulating inflammatory responses [75] and influencing gut motility [76]. Although the mechanism linking EA ingestion to increased TA production is unclear, one possibility is worth noting: Harlow et al. [39] found that hyperammonia-producing bacteria, including various *Clostridium species*, such as *Clostridium sporogenes*, can degrade EV. Within this genus, certain species, like *Cl. Sporogene*, possess decarboxylases, enzymes that convert tyrosine and tryptophan into TAs, suggesting that EA ingestion (at low or high levels) may increase the activity of these microorganisms, potentially leading to increased TA production. Although the abundance of the *Clostridiaceae* family in the rumen remains unchanged here, this does not necessarily mean that these microbes are not more metabolically active. In view of the similar mechanisms of action between TAs and EAs, do trace amines play an important role in the pathophysiology of FT? It may be thus worthwhile to explore the link between TAs dyshomeostasis and FT further.

The presence of an endophyte fungus in tall fescue grass, whether toxic or novel, influenced the animal metabolome, particularly the amino acid level, carbohydrate metabolism, and the biosynthesis of unsaturated fatty acids. In addition, an observed overlap in C18 metabolic features between E+ and NT groups suggests a similar metabolic profile, at least for non-polar compounds. Aerobic microbes, including fungi, are unlikely to thrive in the anaerobic environment of the rumen, making it questionable whether they would trigger a response in the animal that could alter the metabolome or microbiome. On the other hand, the primary distinction between the two endophytes lies in their ability to produce EAs; while the novel endophyte was selected to produce non-toxic levels for animals, its production is not negligible [22], raising the possibility that even minimal EA concentrations could interact with the animal metabolome. Moreover, both strains retain the ability to synthesize anti-insect compounds, such as peramines and lolines alkaloids [21,77]. Although these compounds are considered non-toxic to grazing animals based on signs of FT [78,79], their interaction with the animals’ metabolome has not been studied. All together, these data suggest that the secondary metabolites, rather than the endophyte itself, are likely responsible for the metabolic changes/overlap observed in the “endophyte-effect” group, but future research may provide further clarification.

An interesting finding from this fall-grazing trial we already reported [23] was that steers grazing NT pastures were able to maintain their tail-skin surface temperature (SST) throughout most of this study. Here, we found that three rumen metabolites, lysophosphatidylcholine, decanoylcarnitine, and ornithine, and one urinary metabolite, L-glutamate, were positively correlated with the SST in these animals. Something in common among the rumen metabolites is that they act directly or indirectly at the mitochondrial level, influencing processes such as ATP production and energy efficiency [80,81]. On the other hand, dietary glutamine has been shown to help lower core body temperature during heat stress and increase heat shock protein production [82]. Furthermore, a microinjection of L-glutamate into the preoptic area has been shown to reduce shivering and triggers heat-loss responses, such as cutaneous vasodilation [83], emphasizing its role in thermoregulation. These findings suggest that animals grazing NT pastures may rely on mitochondrial activity and L-glutamate to support better body temperature regulation.

## 4. Conclusions

The data presented here provide evidence that even low levels of EAs, found in endophyte-infected fescue, have a greater impact on the metabolome than on the microbiome, which ultimately can have repercussions on clinical signs. This study, along with the one in which we reported the physiological and behavioral effects of E+ [23], demonstrated that while the levels of EAs were low, their impact on physiological and metabolic processes in the animals was still evident. Our metabolomics findings herein align with the previously published literature, supporting the observed trends. Additionally, the presence of an endophyte in the fescue grass, likely through the production of secondary metabolites, impacted the metabolome of grazing animals, especially the amino acid level, carbohydrate metabolism, and the biosynthesis of unsaturated fatty acids. While these metabolic changes did not lead to clinical alterations in the NT group, the situation was different in the E+ group, in which metabolic shifts, mainly in lipid metabolism, may have complemented or even enhanced the effects of low-level ergot alkaloid ingestion, contributing to disease pathogenesis. This suggests that the endophyte-induced metabolic changes could have exacerbated the negative effects of EAs. Our study also suggests that the potential role of trace amines in the pathophysiology of FT should be studied further.

## 5. Materials and Methods

### 5.1. Animals, Treatments, and Experimental Design

**Figure 5:**
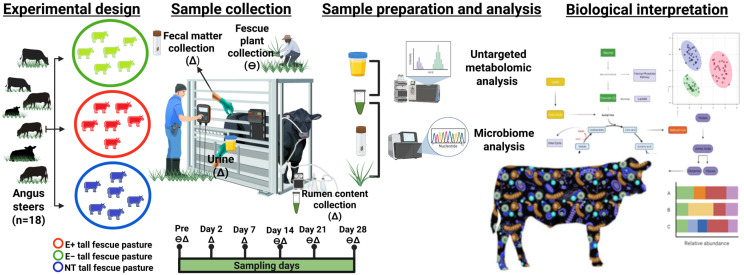
Farm-to-data workflow. Angus steers (n = 18) were placed on toxic endophyte E+ (3 paddocks; 2 steers per paddock), non-toxic endophyte NT (3 paddocks; 2 steers per paddock), and endophyte-free fescue E− (3 paddocks; 2 steers per paddock) throughout a 28-day grazing trial. (∆) denotes days of body weight recording; (θ) denotes urine, feces, and rumen content collection. Untargeted high-resolution metabolomics (HRM) and next-generation sequencing (NGS) for microbiome analyses were performed on these biological samples, followed by a biological interpretation.

This study was conducted in the Fall of November 2021 on pastures located at the University of Georgia’s J. Phil Campbell Natural Resources Conservation Center (Watkinsville, GA, USA) as described in [23]. Briefly, post-weaning steers (n = 18) were separated by weight and randomly assigned to 0.4 ha of non-toxic (weight: 263.1 ± 4.91 kg; NT; Jesup MaxQ with endophyte AR542; 3 paddocks; 2 steers per paddock), toxic (weight: 259 ± 5.05 kg; E+; Jesup with wild-type endophyte; 3 paddocks; 2 steers per paddock), and endophyte-free fescue pastures (weight: 258.9 ± 5.04 kg; E−; 3 paddocks, 2 steers per paddock). The steers were kept on pasture for 28 days. Prior to (pre-) and 2, 7, 14, and 28 days post-pasture assignment, urine, fecal matter, and rumen content (solid and fluid) were collected (Figure 5). Fescue plants were collected on the 1st, day 14, and the last day of the trial for different analyses, as described below.

### 5.2. Sample Collection and Processing

Voided urine samples were collected in sterile cups and placed on ice in 15 mL conical centrifuge tubes (Fisher Scientific, Waltham, MA, USA). Fresh fecal samples were collected by hand using a new glove for each collection, placed in 50 mL conical tubes, and stored on ice (Figure 5). Samples of ruminal content were collected as in [16] using an ororuminal probe, which was washed between use on animals. Approximately 50 mL of ruminal contents was filtered using four layers of autoclaved cheesecloth, which allowed the separation of the rumen liquid from the rumen solids. The collected rumen content was placed in sterile cryovial tubes and kept on dry ice. Upon arrival at the lab, urine samples were immediately centrifuged (300× *g* for 10 min at 4 °C) and aliquoted. All the samples were stored at −80 °C until further analysis.

#### Endophyte Detection and Total Plant Ergot Alkaloids Analysis

Sampling was performed by selecting a tiller from 100 locations within the pastures, cutting the tiller at the soil surface, and transporting the samples to the laboratory. Endophyte presence was analyzed from a 3 mm cross-section of the stem base of each tiller using a commercial immunoblot test kit (Agrinostics Ltd., Co., Watkinsville, GA, USA, Cat. # ENDO797-3). In addition, total plant ergot alkaloid content was determined by a second tiller cross-section using a commercial ELISA test kit (Agrinostics Ltd., Cat. # ENDO899-96p), with a limit of detection (LOD) of 1 ppb, as in [14].

### 5.3. Metabolomics Sample Processing and Data Analysis

#### 5.3.1. Metabolomics Sample Processing

Urine and rumen fluid metabolomics samples were processed as described in [14]. Briefly, 50 μL of urine or rumen fluid was combined with 100 μL acetonitrile and 2.5 μL of an isotopically labeled internal standard mixture containing [trimethyl-^13^C_3_] caffeine, [^15^N,^13^C_5_]-L-methionine, [^15^N]-L-tyrosine, [^13^C_5_]-L-glutamic acid, [^13^C_6_]-D-glucose, [3,3-^13^C_2_]-cystine, [^13^C_18_]-linoleic acid, [3′,4′,5′-^13^C_3_]-nicotine, [^13^C_6_]-L-histidine, and [2,3,4-^13^C_3_]-cortisol. Samples were kept on ice for 30 min before centrifugation at 14,000 rpm for 10 min. Subsequently, 100 μL of the supernatant was analyzed using the High-Field Orbitrap Mass Spectrometer with specific instrument settings [84]. Both reverse-phase (C18) chromatography with negative electrospray ionization (ESI) and hydrophilic liquid interaction chromatography (HILIC) with positive ESI were employed, with each sample run in triplicate for both methods. LC-MS conditions and methodology used were as described previously [84]. Data processing involved various steps, such as peak detection, noise filtering, *m/z* and retention time alignment, feature quantification, and quality filtering, executed using apLCMS v6.1.3 [85] followed by xMSanalyzer v2.0.7 [86]. Data were extracted as *m/z* features, defined by *m/z*, retention time, and integrated ion intensities. Technical replicates were median summarized for subsequent bioinformatics analysis.

#### 5.3.2. Urine and Rumen Fluid Metabolite Annotations

All metabolomics annotations presented here were generated using either the human metabolome database (HMDB); bovine metabolome database (BMDB), livestock metabolome database (LMDB), or the toxic exposome database (T3DB). Significantly different *m/z* features were annotated against databases with Δ 5 ppm tolerance. When the same metabolic feature was detected with different *m/z* values due to multiple co-eluting additional adducts, the feature with the lightest adduct was selected. For the negative ionization mode (C18 column), the following adducts were considered for annotation: M−H, M−H_2_O −H, M + Na−2H, M + Cl, and M + FA−H. For the positive ionization mode (HILIC column), adducts including M + H, M + 2H, M + H + NH_4_, M + ACN + 2H, M + 2ACN + 2H, M + NH_4_, M + Na, M + ACN + H, M + ACN + Na, M + 2ACN + H, 2M + H, 2M + Na, 2M + ACN + H, M + 2Na−H, M + H−H_2_O, and M + H−2H_2_O were considered.

#### 5.3.3. Metabolomics Data Processing and Statistical Analysis

The metabolomic data were preprocessed using MetaboAnalyst 5.0 [87] (https://metaboanalyst.ca, accessed from September to December 2023), where they successfully passed the data integrity check. Data were filtered utilizing the interquartile range (IQR) method to eliminate noise and non-informative variables. Moreover, the datasets were filtered based on the relative standard deviation (RSD/mean) to remove the features with low repeatability. Subsequently, normalization was conducted by quantile normalization [88], ensuring uniformity in the total ion peak area across samples. Additionally, log10 transformation was applied to better fit the distribution characteristics of the data, followed by Pareto scaling. Univariate and multivariate analyses were performed using metabolic features extracted from HILIC and C18 columns for rumen fluid and urine samples. A multivariate analysis using sparse partial least squares discriminant analysis (sPLS-DA) was performed to identify the features with the strongest discriminatory power among the different treatment groups. To further investigate the metabolic features most influenced by the effect of E+ toxin(s), multiple comparisons were conducted using two-sample *t*-tests (*p* ≤ 0.05). Comparisons were made between E− and E+, E− and NT, and NT and E+ groups. To address the issue of multiple comparisons, the Benjamini–Hochberg false discovery rate was employed (FDR; FDR = 0.05). Table 3 shows how the significant detected features were considered for E+ or an endophyte effect. Features that were most affected by the E+ toxin(s), as well as those identified by sPLS-DA as having the greatest discriminatory power between the E+ group and other groups, were analyzed further. A two-way repeated measures analysis of variance (ANOVA) followed by Tukey’s honestly significant difference (HSD) post hoc test was used to assess how these features fluctuated across the sampling days. Additionally, these features were correlated with physiological parameters, such as weight gain, respiration rate, rectal temperature, and skin surface temperature, as reported in Table 3. Significant correlations were identified using Kendall’s rank correlation (*r* ≥ 0.4; *p* ≤ 0.05).

For the entire grazing trial, metabolic pathway analysis was conducted on the ruminal fluid and urine high-resolution metabolomics features using mummichog 2.0 [87], and annotated with the *Bos taurus* KEGG database [89]. Features that differed between groups (*p* ≤ 0.05, FDR ≤ 0.2) were selected for the analysis. The metabolic pathways reported in this study are those influenced by the presence of the endophyte and the effects of E+ toxin(s), and their selection was based on the criteria outlined in Table 3. Their significance is shown by the −log10 mummichog corrected *p*-value (Figure 3).

### 5.4. Microbiome Sample Processing and Data Analysis

#### 5.4.1. DNA Extraction

For the fecal and rumen content (solid and fluid), genomic DNA extraction was performed using a modified protocol established by [90], employing mechanical disruption and phenol extraction with a 25:24:1 phenol:chloroform:isoamyl alcohol ratio [91]. Following extraction, all DNA samples were resuspended in TE (Tris-EDTA) buffer and quantified using a Qubit^®^ Fluorometer (Invitrogen, San Diego, CA, USA). To ensure the reliability of the procedure, a negative control (TE extraction buffer) was included alongside each extraction and subjected to the same amplification and sequencing protocols outlined below. Tall fescue samples were stomached for 5 min in a sterile stomacher bag with TE extraction buffer. The supernatant was collected and subjected to the same extraction procedures as previously described [15,48].

#### 5.4.2. DNA Amplification and Sequencing

The sequencing of the bacterial 16S rRNA gene was performed as described in [15,48]. Briefly, samples were diluted to 1 ng/μL for amplification, employing universal bacterial primers targeting the 16S rRNA gene variable region V4 [92]. Negative PCR controls, containing extraction buffer and primers, were included. Each 25 μL reaction mixture comprised 5 μL diluted DNA, 0.5 μL each of 10 μM forward (5′-GTGCCAGCMGCCGCGGTAA-3′) and reverse primers (5′-GGACTACHVGGGTWTCTAAT-3′), 6.5 μL water, and 12.5 μL KAPA HiFi master mix (Kapa Biosystems, Wilmington, MA). Cycling conditions included initial denaturation at 95 °C for 3 min, followed by 25 cycles of 95 °C for 30 s, 55 °C for 30 s, 72 °C for 30 s, and a final extension at 72 °C for 5 min. PCR products underwent purification using 1% (wt/vol) low-melt agarose gel and a Zymoclean 96-well DNA recovery kit (Zymo Research, Irvine, CA, USA). Samples with visible bands in negative controls were reprocessed until no bands were observed. Quantification was performed using a Qubit fluorometer, and samples were equimolarly pooled into the final library. Sequencing was conducted on an Illumina MiSeq platform using a 2 × 250-bp paired-end MiSeq v2 sequencing kit (Illumina, San Diego, CA, USA) with custom primers [92]. Quality filtering and normalization procedures were applied to control and sample DNA prior to further analysis. Sequencing of the archaeal 16S rRNA gene was performed by amplifying the V6–V8 region using a two-step primer approach that included archaeal-specific primers (Ar915aF-AGGAATTGGCGGGGGAGC AC, Ar1386R-GCGGTGTGTGCAAGGAGC) as described by [93], and the subsequent procedure was the same as that described for bacteria.

#### 5.4.3. 16S rRNA Gene Sequence Processing and Bioinformatics Analysis

Raw sequence files were obtained in FASTQ format from the sequencer and processed using Qiime v.2023.2. Paired-end sequences were imported into QIIME 2 [94], followed by the removal of non-biological nucleotides and subsequent denoising, dereplication, and chimera-filtering utilizing DADA2 [95]. After this preprocessing, sequences were clustered into Amplicon Sequence Variants (ASVs) at a 100% identity; similarity threshold and taxonomic classification were performed employing the Silva 138 small subunit (SSU) database [96]. A pre-trained naive Bayes classifier, trained on the SILVA 138 SSU database, was used to assign taxonomies to the sequences (Silva210_475_q20232).

Each dataset was analyzed separately. Before assessing both alpha and beta diversities using the “qiime diversity” plugin, the samples were rarified to 12,300, 8000, 7500, and 300 sequences per sample of rumen fluid, solid, feces, and fescue plant, respectively. These specific values were chosen to ensure a minimum Good’s coverage index of 0.99 for all the matrices, except for the plant material, which had a coverage index of 0.96. Alpha-diversity metrics, including Shannon diversity index, Faith’s Phylogenetic Diversity, Pielou’s evenness, and the number of observed features were computed, and their significance was assessed using two-way repeated analysis of variance (ANOVA) to evaluate differences between treatments, days, or treatment–day interactions. Beta-diversity of bacterial communities was determined using Venn diagrams to visualize unique ASVs that differentiated groups, and Unweighted UniFrac distances with 999 permutations, which were visualized in 2-dimensional plots with EMPeor [97]. Venn diagrams were built with ASVs that represented ≥0.1% of the total community within each of the microbial groups. Microbial taxa with relative abundances ≥ than 0.1% in fescue plant and 0.4% in the other matrices were analyzed for group differences using the Kruskal–Wallis test (*p* ≤ 0.05), followed by Dunn’s post hoc analysis with the Bonferroni correction. The findings were considered significant at *p* ≤ 0.05, with *p*-values between 0.05 and 0.10 treated as trends. Microbial taxa that were statistically significant between E+ and the other groups were correlated with physiological parameters (weight gain, respiration rate, rectal temperature, skin surface temperature), as well as metabolic features influenced by E+ toxin(s) found in urine and rumen fluid, using Kendall’s rank correlation (r > 0.3; *p* < 0.05).

For Archaea, the resulting sequences were processed using mothur v.1.44.0 [98], as previously described [91]. Briefly, paired-end sequences were combined into contigs and poor-quality sequences were removed. Sequences were subsequently aligned against the SILVA 16S rRNA gene database and contigs that did not align were removed. Sequences were pre-clustered to reduce sequencing errors and chimera detection, and removal was performed. The sequences were classified to the SILVA (v138.1) database and assigned to OTUs at a 97% sequence similarity. Sample coverage was assessed by Good’s index [99] in mothur. Finally, samples were normalized by total group (relative abundance × lowest number of sequences per sample). The same statistical test and criteria used for bacteria were applied to examine differences in archaeal relative abundance at the genus level. The only difference was that no abundance threshold was applied for Archaea.

## Figures and Tables

**Table 1:** Metabolic features that exhibited discriminatory power in the sPLSDA analysis; were influenced by the effect of E+ toxin(s); and showed differences (*p* ≤ 0.05) post- but not pre-pasture placement in Angus steers grazing endophyte-infected (E+) throughout the 28-day study period.

RUMEN FLUID				
***m/z***	**Retention Time (s)**	**Annotation**	**Adducts**	**Database**
206.1286	34	Kynuramine	M + ACN + H	BMDB0012246
185.1285	43	**Methoxytyramine**	M + NH4	BMDB0012162
128.107	39	Iso-Valeraldehyde	M + ACN + H	BMDB0063681
169.1335	42	**Methyltyramine**	M + NH4	BMDB0003633
183.1129	47	Phenylalanine	M + NH4	BMDB0000159
143.1180	69	Isopropyl alcohol	M + 2ACN + H	HMDB00863
213.1597	36	14-Bipiperidine-1-carboxylic acid	M + H	HMDB60336
249.1346	35	Histidylproline diketopiperazine	M + H	HMDB02053
155.1179	39	**Tyramine**	M + NH4	BMDB0000306
197.1285	40	**Salsolinol**	M + NH4	BMDB0096156
112.0757	38	**3,4-Dimethoxyphenylethylamine (DMPEA)**	M + ACN + H	BMDB0096131
313.1069	31	Not identified		
251.0713	187	7-hydroxy-2-methylisoflavone	[M-H]^-^	HMDB33979
316.2572	224	Monoacylglycerol (MG-0:015:00)	[M-H]^-^	HMDB11532
357.2015	251	5-HETE	[M + Cl]^-^	C04805
**URINE**				
155.1179	37	**Tyramine**	M + NH4	BMDB0000306
169.1336	35	**Methyltyramine**	M + NH4	BMDB0003633
171.1128	67	Dopamine	M + NH4	BMDB0000073
185.1285	89	**Methoxytyramine**	M + NH4	BMDB0012162
197.1285	40	**Salsolinol**	M + NH4	BMDB0096156
209.0688	62	1H-Indole-3-carboxaldehyde (metabolite of L-tryptophan)	M + ACN + Na	BMDB0063668 and LMDB00661
213.1233	64	Acetyldopamine	M + NH4	BMDB0096146
268.1764	41	Histidylleucine	M + NH4	BMDB0063926
112.0757	42	**3,4-Dimethoxyphenylethylamine (DMPEA)**	M + ACN + 2H	BMDB0096131
173.1172	66	4-Hydroperoxy-2-nonenal	M + H	HMDB6027
213.0114	144	Not identified		
214.1265	64	7a,12a-Dihydroxy-3-oxo-4-cholenoic acid	M + H + Na	BMDB0000447
246.1813	80	Lysyl-Valine	M + H	HMDB28964
284.1716	38	Histidinyl-Lysine	M + H	HMDB28890

Annotations include references from the BMDB (bovine metabolome database), HMDB (human metabolome database), LMDB (livestock metabolome database), and KEGG. Bolded annotations are shared across both matrices.

**Table 2:** Correlation analyses summary for each matrix. Kendall’s rank correlations between significant bacteria and archaea with E+-influenced metabolic features and physiological parameters, as well as between key E+-related metabolic features found in urine and rumen fluid with physiological parameters.

Matrices	Family/Genera	Metabolic Feature *m/z*	rt (s)	Annotation	Phys. Param	Kendall’s Rank	*p*-Value
Rumen fluid	*Prevotellaceae*	185.1285 (R)	43	Methoxytyramine	NO	0.54	<0.01
		169.1335 (R)	42	Methyltyramine		0.45	<0.01
		183.1129 (R)	47	Phenylalanine		0.52	<0.01
		197.1285 (R)	40	Salsolinol		0.54	<0.01
						
	*Methanobrevibacter*	185.1285 (R)	43	Methoxytyramine	NO	−0.38	0.02
						
	*Methanomethylophilaceae_uncultured*	185.1285 (R)	43	Methoxytyramine	NO	0.41	0.02
						
		155.1179 (R)	39	Tyramine	RT	−0.41	0.003
		316.2572 (R)	224	Monoacylglycerol	RT	0.39	0.004
							
Rumen solid	*Eubacterium_* *coprostanoligenes_group*	206.1286 (R)	34	Kynuramine	NO	−0.38	0.003
							
Feces	*Rikenellaceae_RC9_gut_* *group*	155.1179 (U)	37	Tyramine	NO	−0.32	0.04
						
	*Clostridium_sensu_* *stricto_1*	112.0757 (U)	42	3,4-Dimethoxyphenylethylamine	NO	−0.41	0.003
		155.1179 (U)	37	Tyramine		−0.39	0.003
		169.1336 (U)	35	Methyltyramine		−0.40	0.002
		185.1285 (U)	89	Methoxytyramine		−0.43	0.001
		197.1285 (U)	40	Salsolinol		−0.40	0.006
							
Urine		112.0757 (U)	42	3,4-Dimethoxyphenylethylamine	WG	−0.59	0.001
		197.1285 (U)	40	Salsolinol	WG	−0.45	0.009

Kendall’s rank correlation coefficients (ranging from −1 to 1) were calculated, with *p*-values indicating statistical significance (≤0.05). NO indicates no significant correlation with physiological parameters; RT refers to rectal temperature; WG refers to weight gain. In the “Metabolic Feature” column, (R) represents metabolites from rumen fluid, and (U) represents those from urine.

**Table 3:** Selection criteria of features and metabolic pathways influenced by the E+ toxin(s) or endophyte effect. For each comparison, “√” indicates significant differences, while “X” indicates no significant differences for a metabolic feature.

E− vs. E+	E− vs. NT	E+ vs. NT	
√	X	√	E+ Toxin(s) effect
X	X	√	
√	X	X	
√	√	X	Endophyte effect
X	√	X	

## Data Availability

The original contributions presented in this study are included in this article and Supplementary Materials. Further inquiries can be directed to the corresponding author.

## Supplementary Materials

The following supporting information can be downloaded at: https://www.mdpi.com/article/10.3390/toxins17050251/s1. The Supplementary Materials file consists of Supplementary File S1: (i) Supplementary Figure S1: extracted ion chromatograms from metabolic features of rumen fluid presented in Table 1; (ii) Supplemental Figure S2: Representative extracted ion chromatograms from metabolic features of urine presented in Table 1; (iii) Supplemental Figure S3: Representative extracted ion chromatograms from metabolic features associated with skin surface temperature; and Supplementary File S2: (iv) Supplementary Table S1. Shannon diversity index, Faith’s phylogenetic diversity index, species evenness, and number of observed features detected in the ruminal content, and feces of Angus steers grazing toxic endophyte-infected (E+; n = 6), non-toxic endophyte-infected (NT; n = 6), and endophyte-free tall fescue E− (E−; n = 6), as well as in the fescue plants themselves (3 paddocks/treatment); (v) Supplementary Table S2. The relative abundance (%) of the main families, genera, and Archaea in the rumen fluid, solid, and feces that significantly changed in steers grazing on tall fescue infected with toxic endophyte (E+; n = 6), compared with steers grazing non-toxic endophyte (NT; n = 6) and endophyte-free (E−; n = 6) grass, over the 28-day study period; (vi) Supplementary Figure S4. Principal coordinate analysis plot of beta diversity (unweighted UniFrac) of bacterial population in the ruminal fluid (A), solid (B), and fecal (C) samples of steers (n = 18) grazing toxic endophyte-infected tall fescue (E+; n = 6), non-toxic endophyte-infected tall fescue (NT; n = 6), and endophyte-free tall fescue E− (E−; n = 6); (vii) Supplementary Figure S5. Relative abundance by day of sampling for selected bacterial and archaeal taxa that showed an overall effect in feces (column A) and rumen fluid (column B).
