# Supplementary material for: Impact of Low-Level Ergot Alkaloids and Endophyte Presence in Tall Fescue Grass on the Metabolome and Microbiome of Fall-Grazing Steers
Source: Toxins (Basel). 2025 May 17;17(5):251. doi: 10.3390/toxins17050251 (PMC12115782; doi:10.3390/toxins17050251)
Supplement: Supplementary file 1 [file toxins-17-00251-s001.zip › toxins-3582876_Supplementary File 1.pdf]

Impact of low-level ergot alkaloids and endophyte presence in tall fescue grass on the metabolome and microbiome of fall grazing steers

Ignacio M. Llada, Jeferson M. Lourenco, M. Mikayla Dycus, Jessica M. Carpente, Garret Suen, Nicholas S. Hill, Nikolay M. Filipov

Supplemental Figure S1: Representative extracted ion chromatograms from metabolic features of rumen fluid presented in Table 1. In all figures, the extracted feature resulted from the major peak present, unless noted otherwise with a black arrow. Retention times reflected may deviate slightly (± 10 s) from extracted features due to feature alignment across samples during untargeted extraction. The first twelve chromatograms resulted from HILIC chromatography with positive electrospray, while the final three chromatograms resulted from reverse phase/negative electrospray.

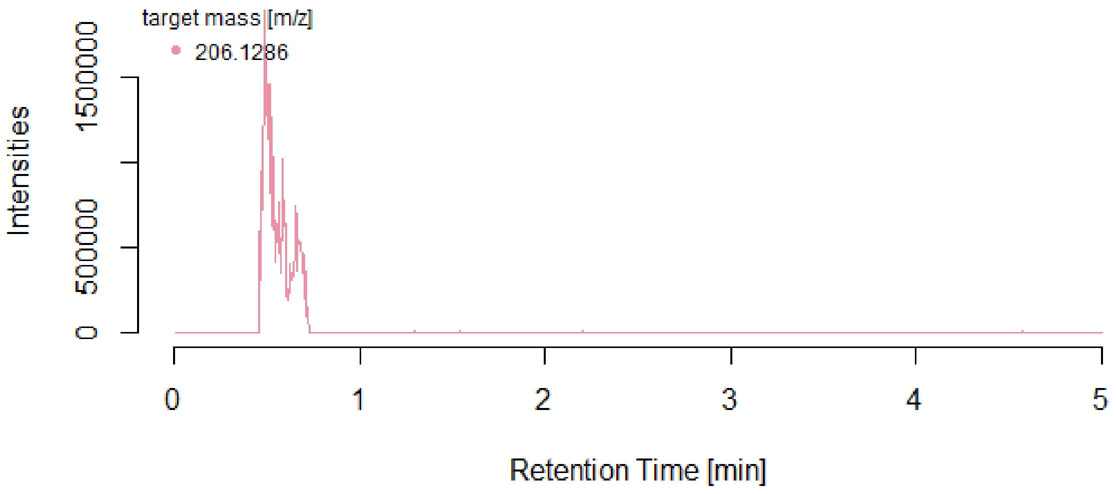

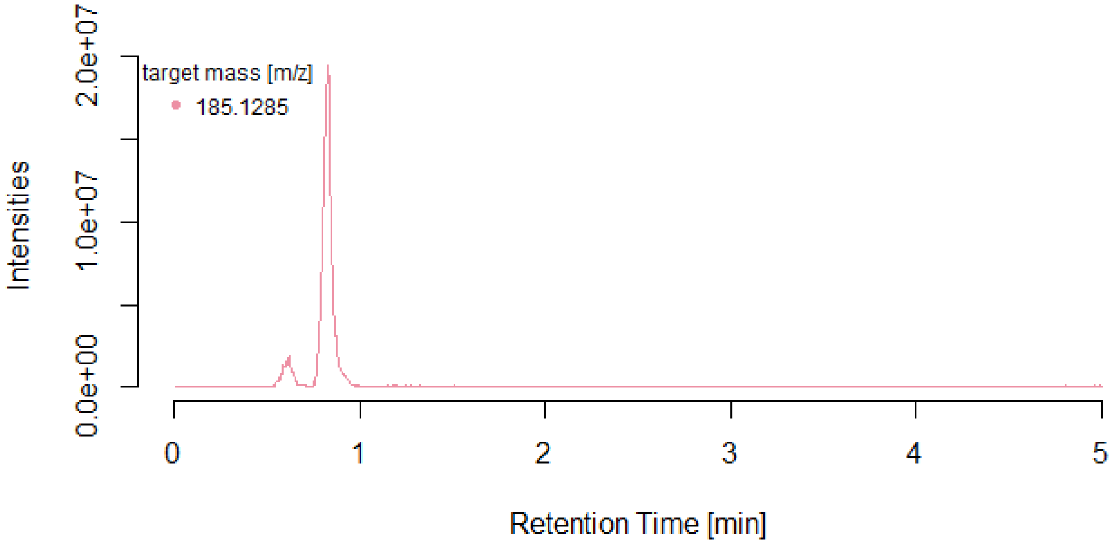

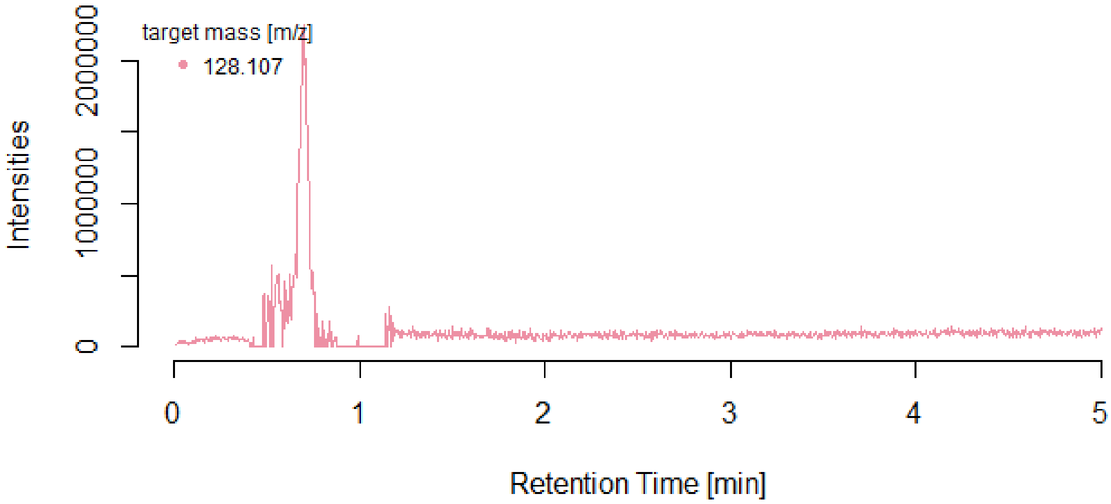
target mass [m/z]
128.107
Intensities
0
1000000
2000000
0 1 2 3 4 5
Retention Time [min]

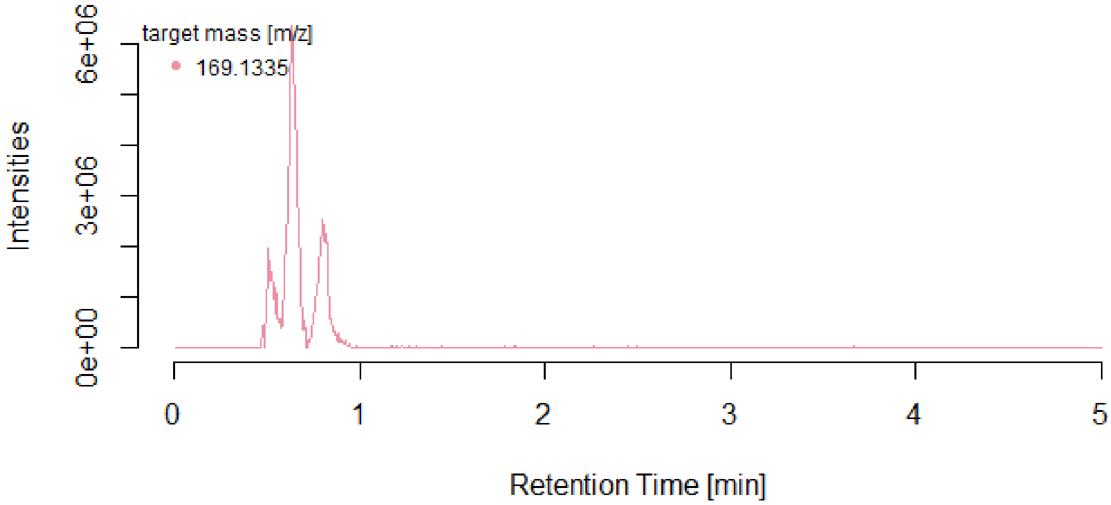
target mass [m/z]
169.1335
Intensities
0e+00
3e+06
6e+06
0 1 2 3 4 5
Retention Time [min]

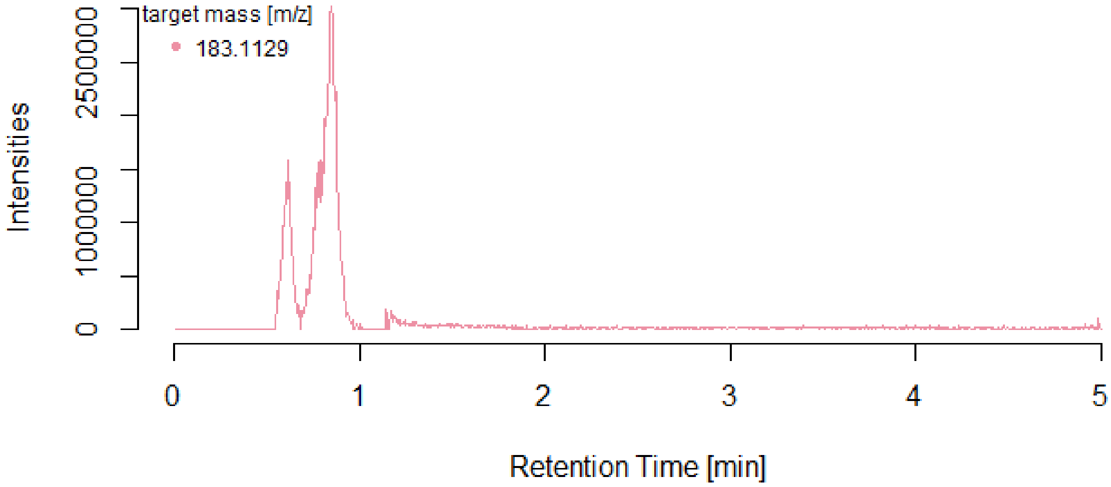
target mass [m/z]
183.1129
Intensities
0
1000000
2500000
0 1 2 3 4 5
Retention Time [min]

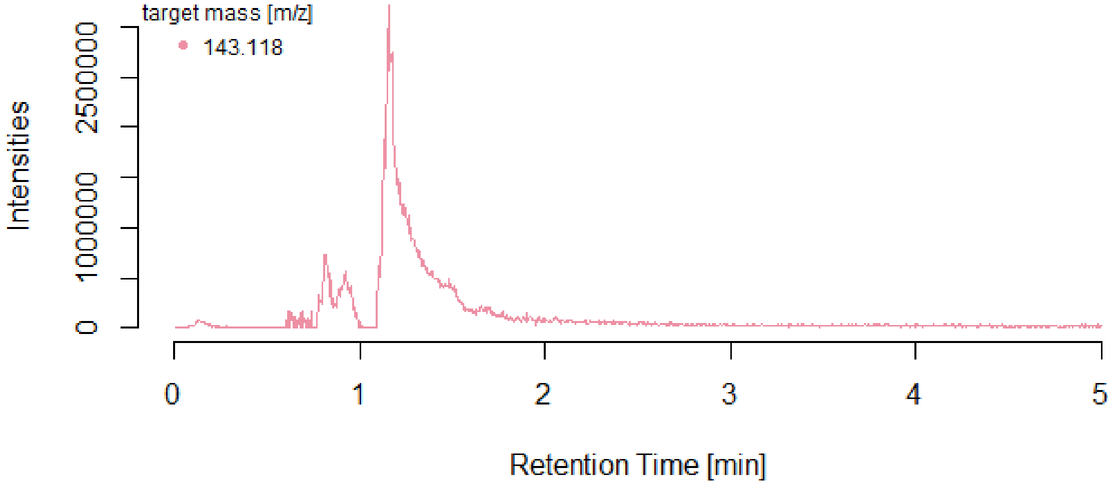
target mass [m/z]
143.118
Intensities
0
1000000
2500000
0
1
2
3
4
5
Retention Time [min]

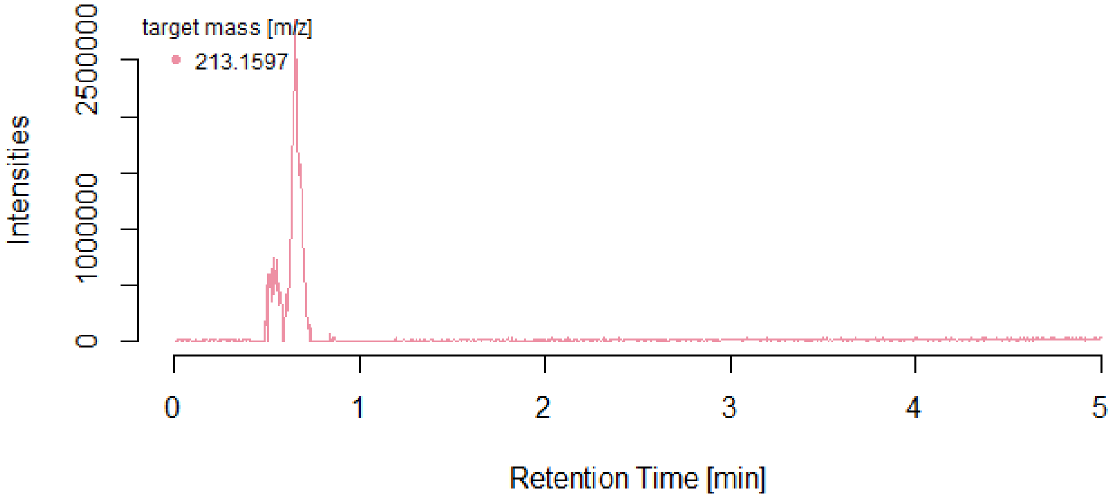
target mass [m/z]
213.1597
Intensities
0
1000000
2500000
0
1
2
3
4
5
Retention Time [min]

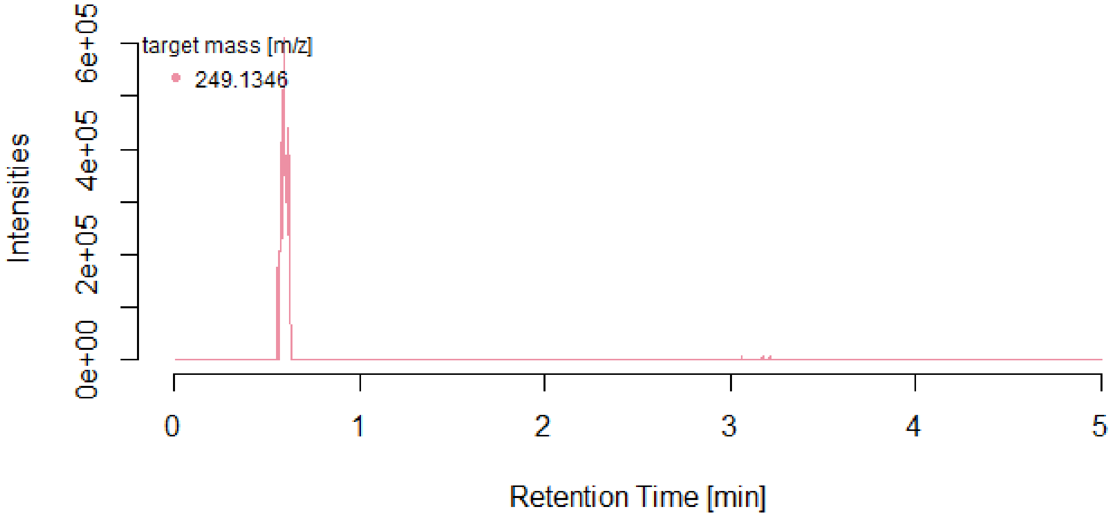
target mass [m/z]
249.1346
Intensities
0e+00
2e+05
4e+05
6e+05
0
1
2
3
4
5
Retention Time [min]

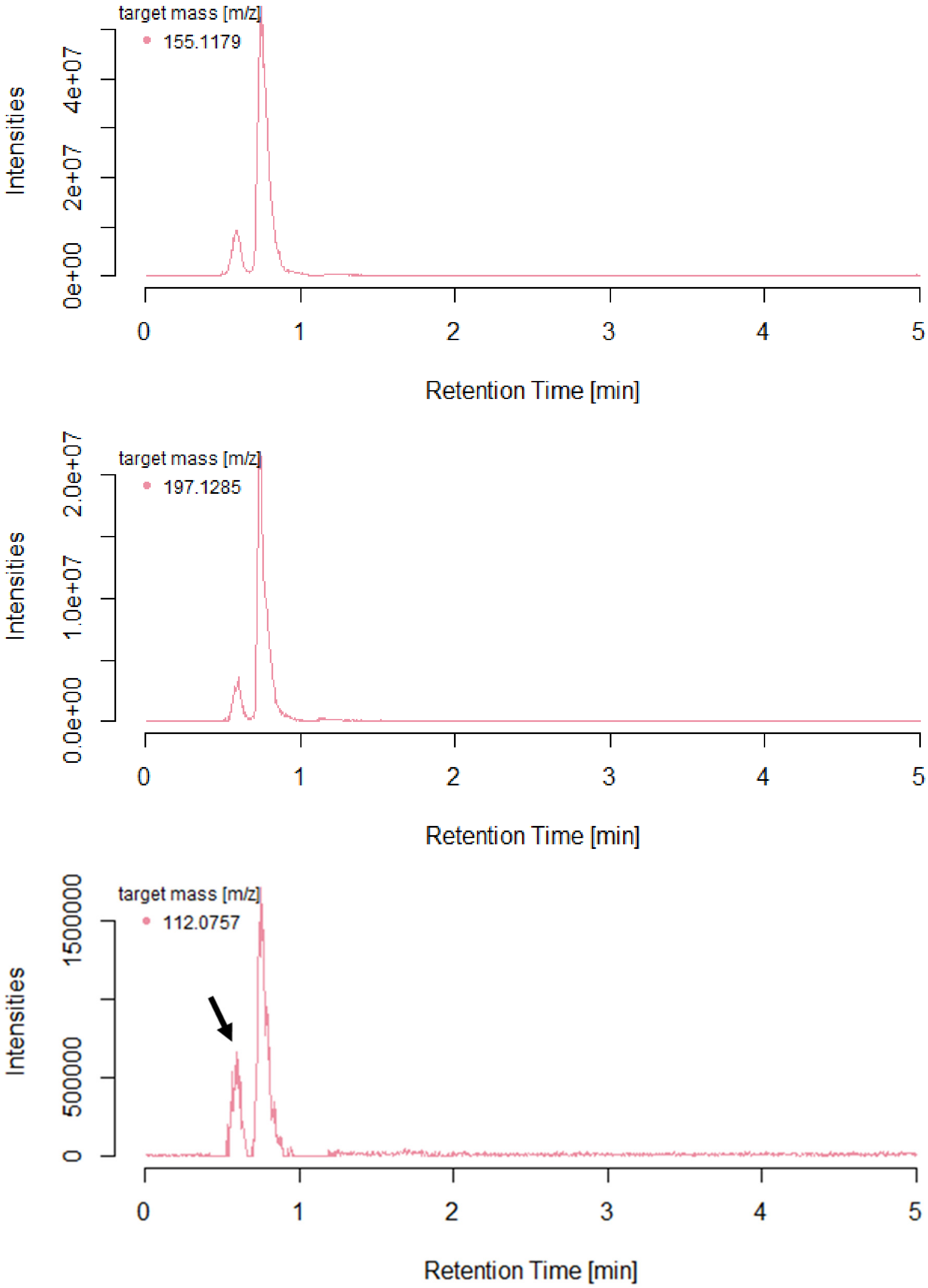

target mass [m/z]
155.1179
Intensities
0e+00
2e+07
4e+07
0
1
2
3
4
5
Retention Time [min]
target mass [m/z]
197.1285
Intensities
0.0e+00
1.0e+07
2.0e+07
0
1
2
3
4
5
Retention Time [min]
target mass [m/z]
112.0757
Intensities
0
500000
1500000
0
1
2
3
4
5
Retention Time [min]

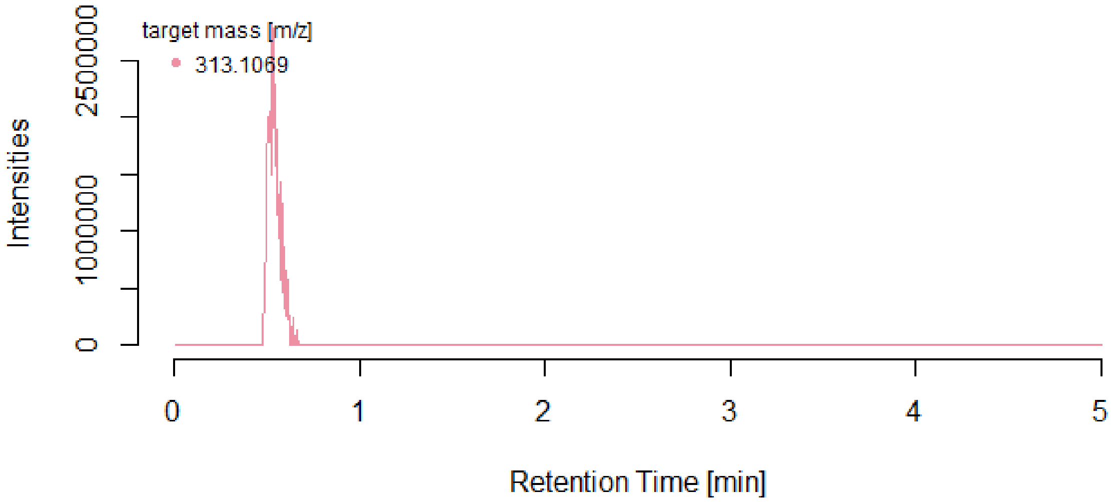
target mass [m/z]
313.1069
Intensities
0
1000000
2500000
0
1
2
3
4
5
Retention Time [min]

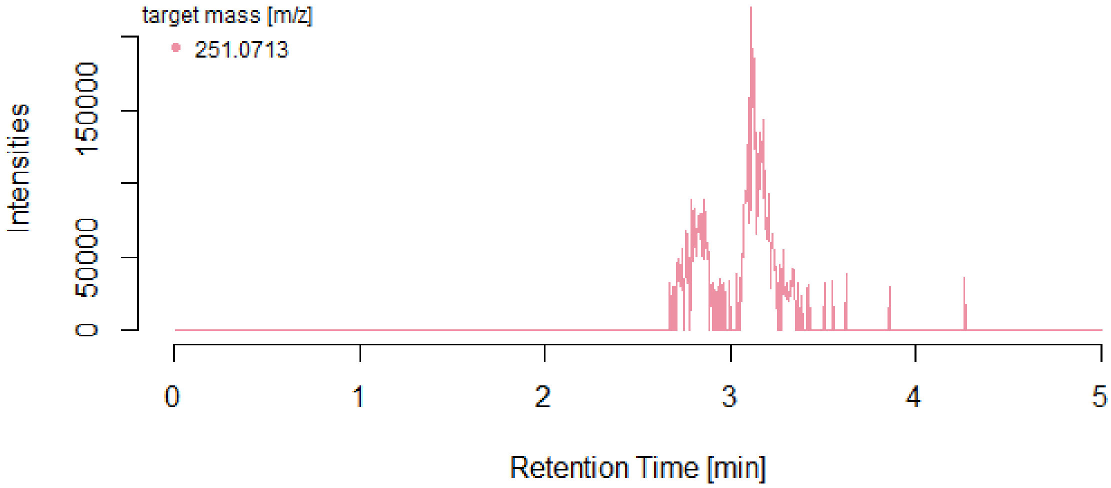
target mass [m/z]
251.0713
Intensities
0
50000
150000
0
1
2
3
4
5
Retention Time [min]

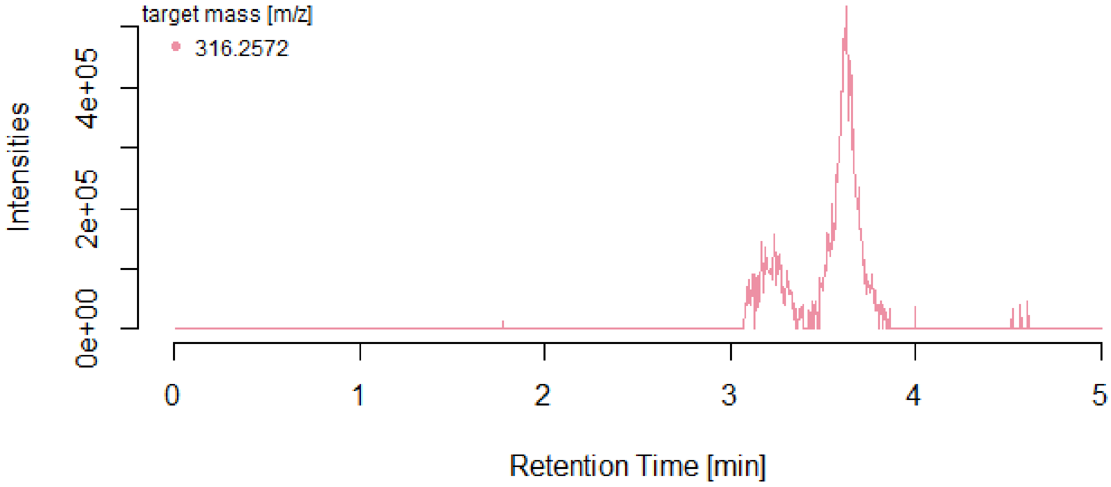
target mass [m/z]
316.2572
Intensities
0e+00
2e+05
4e+05
0
1
2
3
4
5
Retention Time [min]

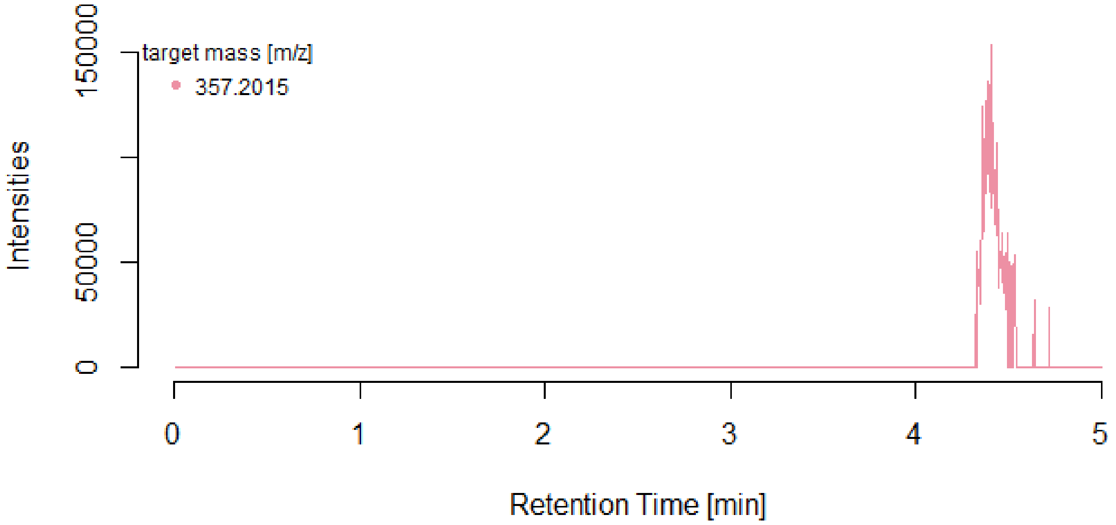

target mass [m/z]
357.2015
Intensities
0
50000
150000
0
1
2
3
4
5
Retention Time [min]

Supplemental Figure S2: Representative extracted ion chromatograms from metabolic features of urine presented in Table 1. In all figures, the extracted feature resulted from the major peak present, unless noted otherwise with a black arrow. Retention times reflected may deviate slightly (± 10 s) from extracted features due to feature alignment across samples during untargeted extraction. The all displayed chromatograms resulted from HILIC chromatography with positive electrospray.

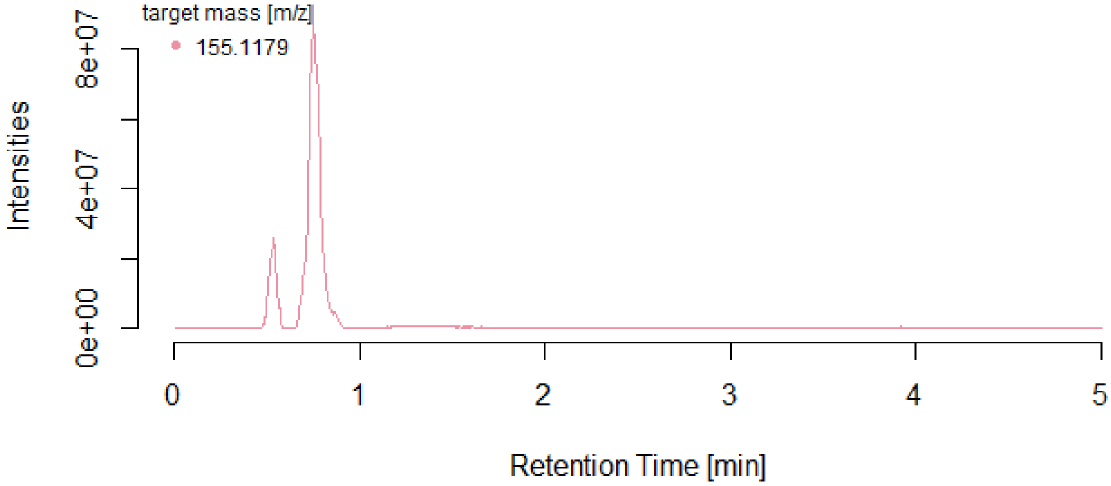

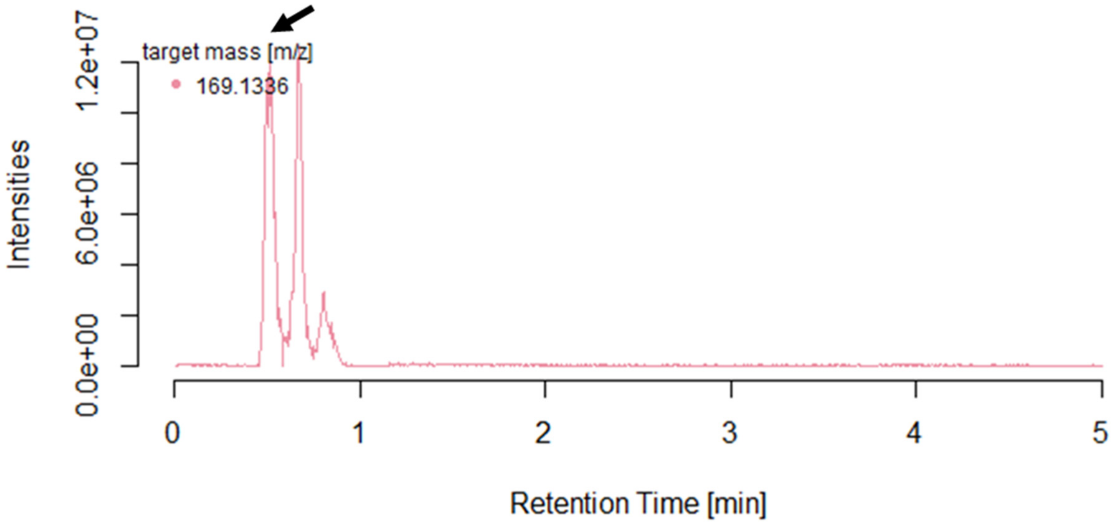

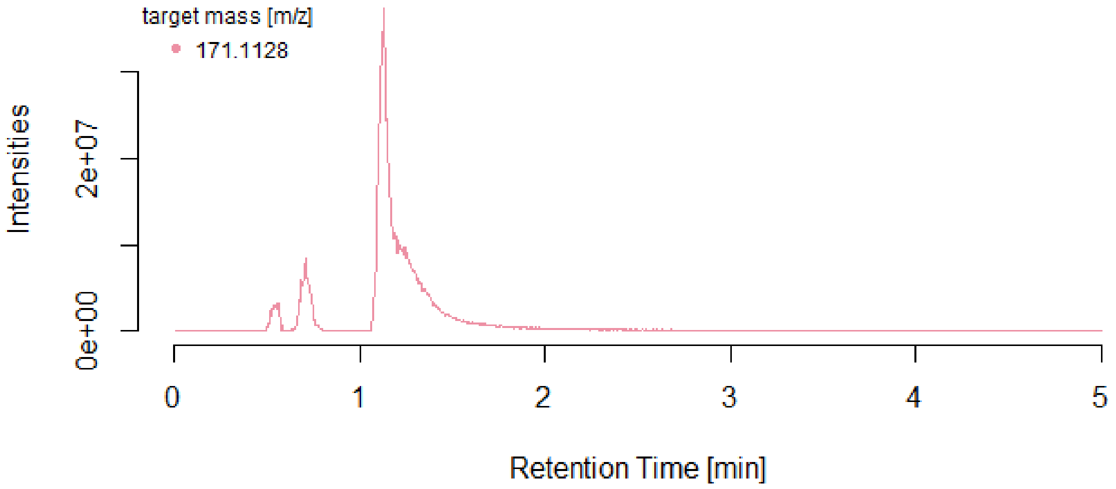
target mass [m/z]
171.1128
Intensities
0e+00
2e+07
0
1
2
3
4
5
Retention Time [min]

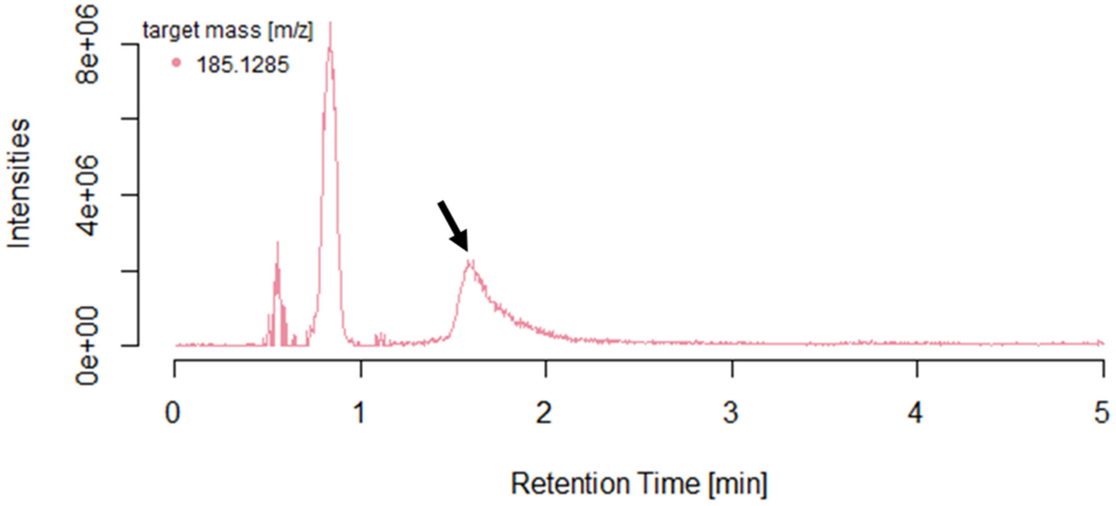
target mass [m/z]
185.1285
Intensities
0e+00
4e+06
8e+06
0
1
2
3
4
5
Retention Time [min]

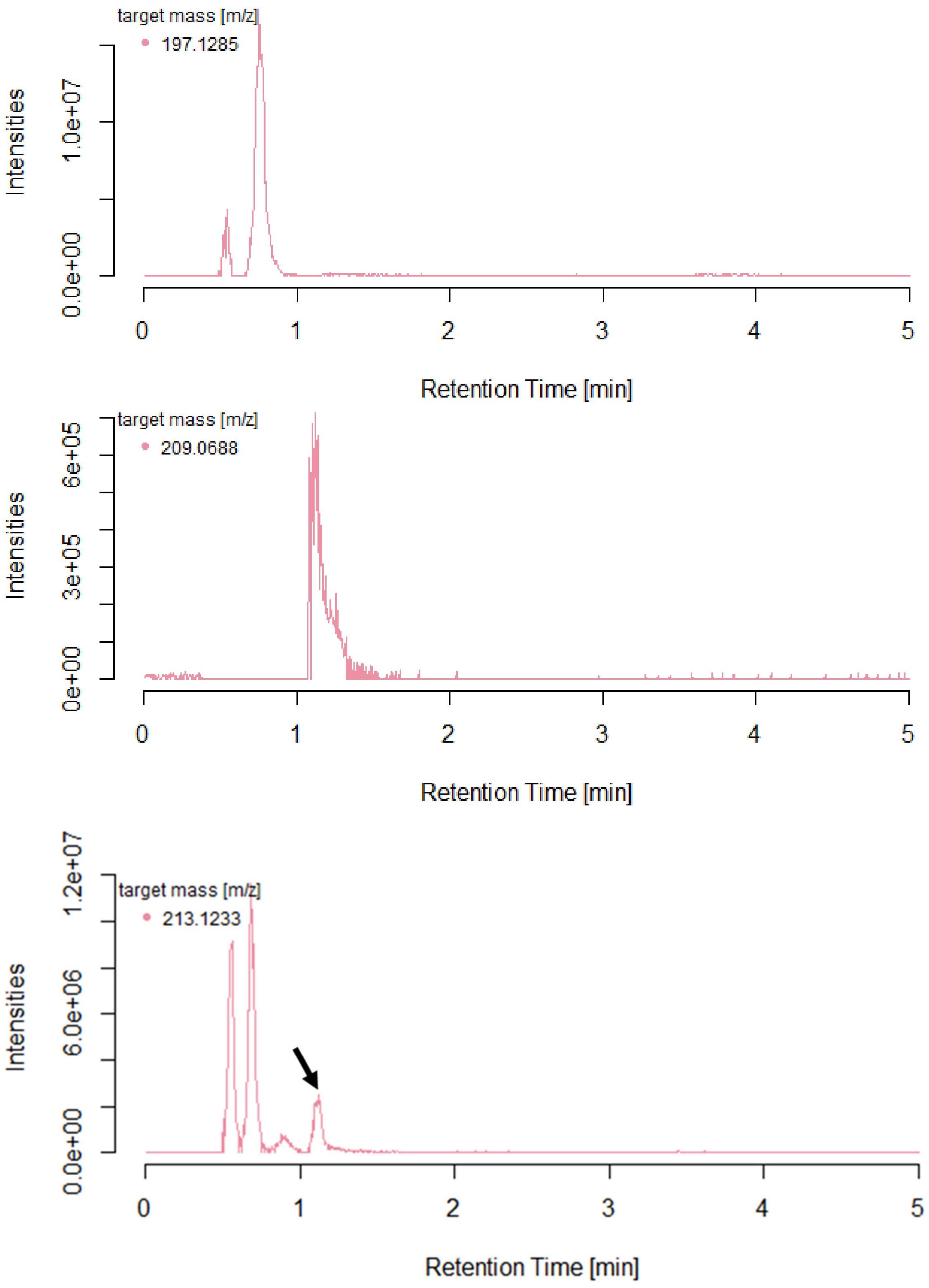
target mass [m/z]
197.1285
Intensities
0.0e+00
1.0e+07
0
1
2
3
4
5
Retention Time [min]
target mass [m/z]
209.0688
Intensities
0e+00
3e+05
6e+05
0
1
2
3
4
5
Retention Time [min]
target mass [m/z]
213.1233
Intensities
0.0e+00
6.0e+06
1.2e+07
0
1
2
3
4
5
Retention Time [min]

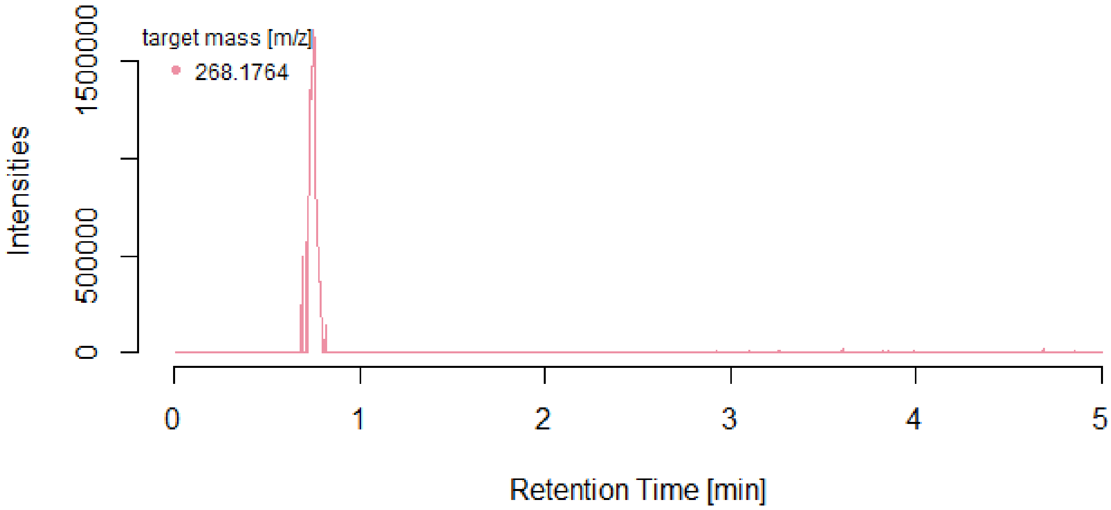
target mass [m/z]
268.1764
Intensities
0
500000
1500000
0
1
2
3
4
5
Retention Time [min]

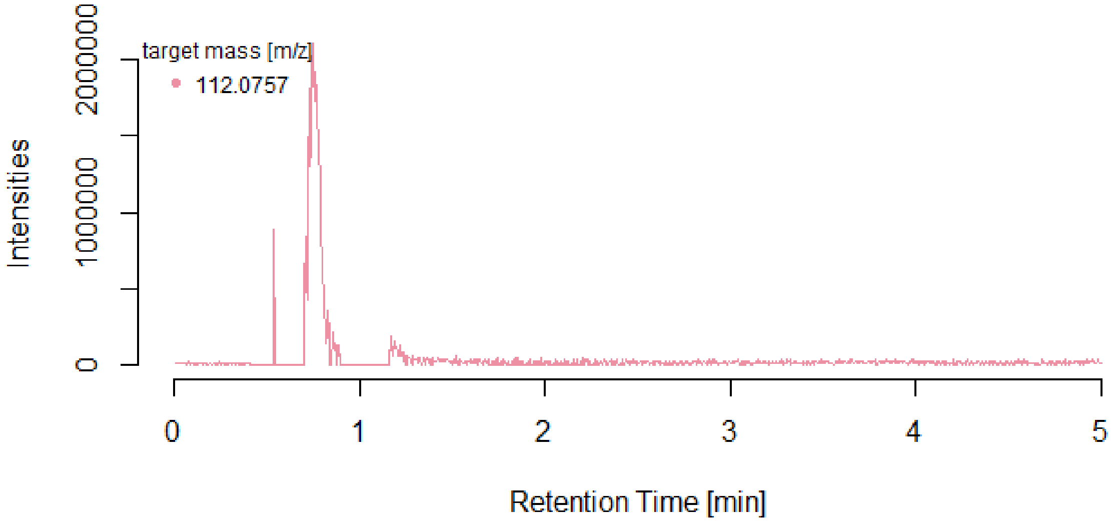
target mass [m/z]
112.0757
Intensities
0
1000000
2000000
0
1
2
3
4
5
Retention Time [min]

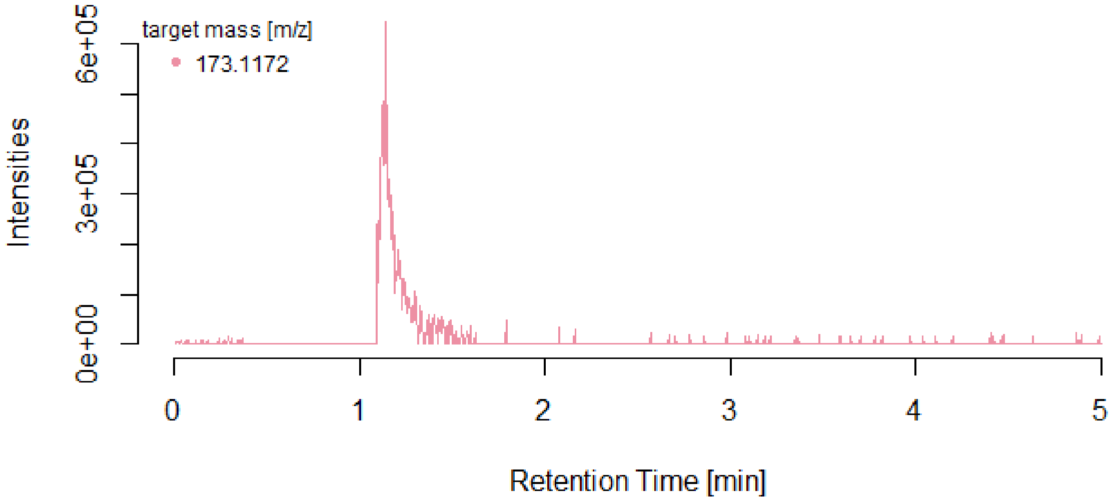
target mass [m/z]
173.1172
Intensities
0e+00
3e+05
6e+05
0
1
2
3
4
5
Retention Time [min]

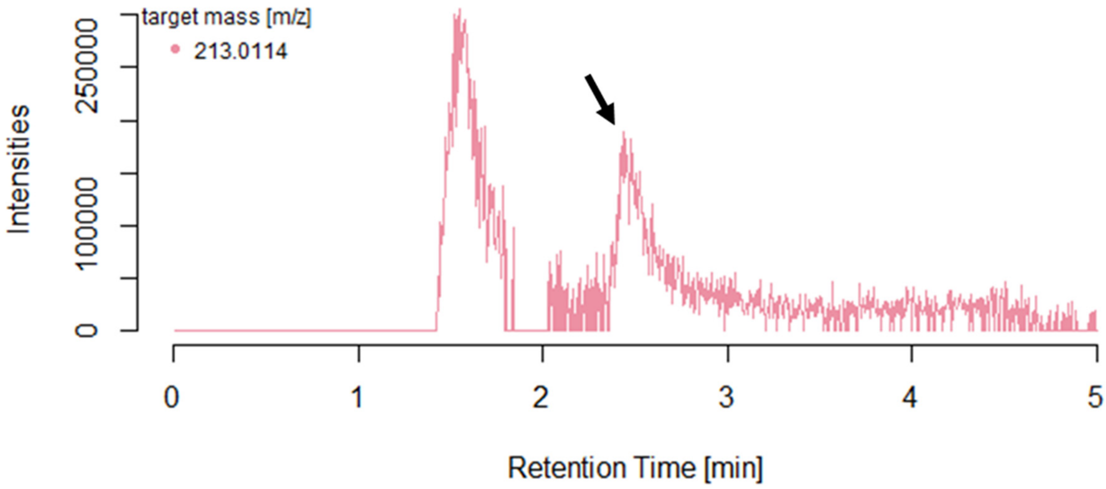
target mass [m/z]
213.0114
Intensities
0
100000
250000
0
1
2
3
4
5
Retention Time [min]

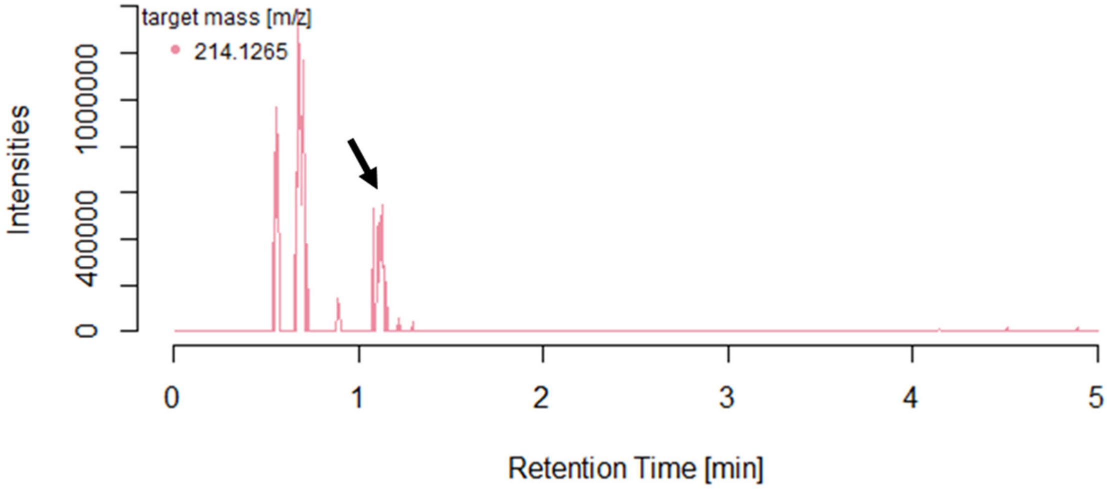
target mass [m/z]
214.1265
Intensities
0
400000
1000000
0
1
2
3
4
5
Retention Time [min]

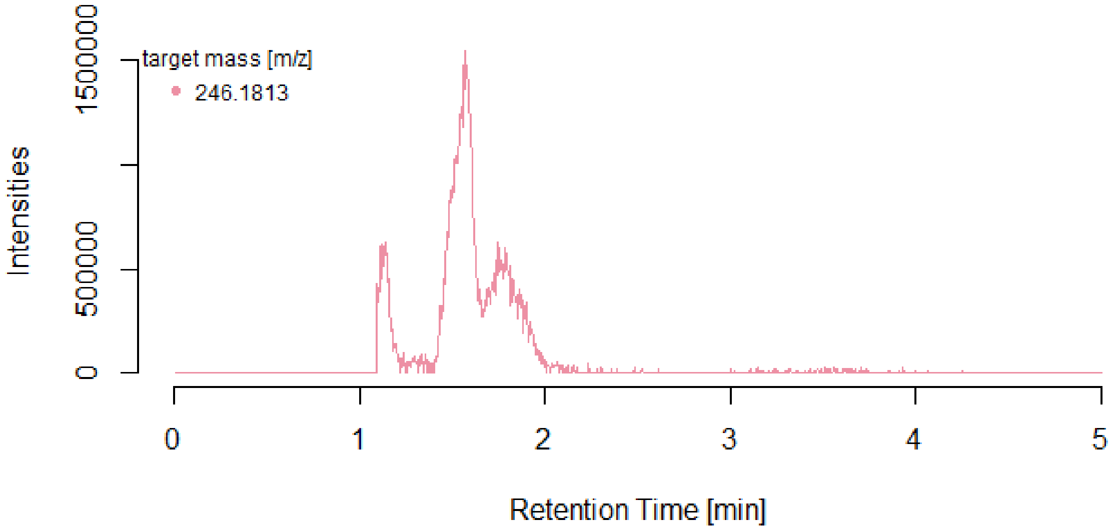
target mass [m/z]
246.1813
Intensities
0
500000
1500000
0
1
2
3
4
5
Retention Time [min]

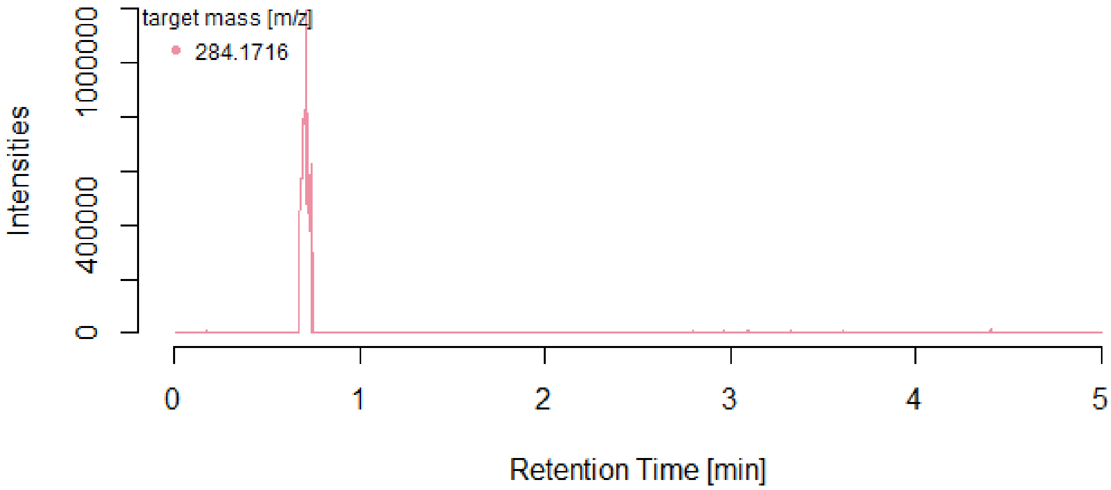
target mass [m/z]
284.1716
Intensities
0
400000
1000000
0
1
2
3
4
5
Retention Time [min]

Supplemental Figure S3. Representative extracted ion chromatograms from metabolic features associated with skin surface temperature. Retention times reflected may deviate slightly (± 10 s) from extracted features due to feature alignment across samples during untargeted extraction. The first three chromatograms are from ruminal fluid samples, with the first chromatogram resulting from HILIC chromatography with positive electrospray, while the second and third chromatogram resulted from reverse phase/negative electrospray. The fourth chromatogram is from urine samples and used HILIC/positive electrospray.

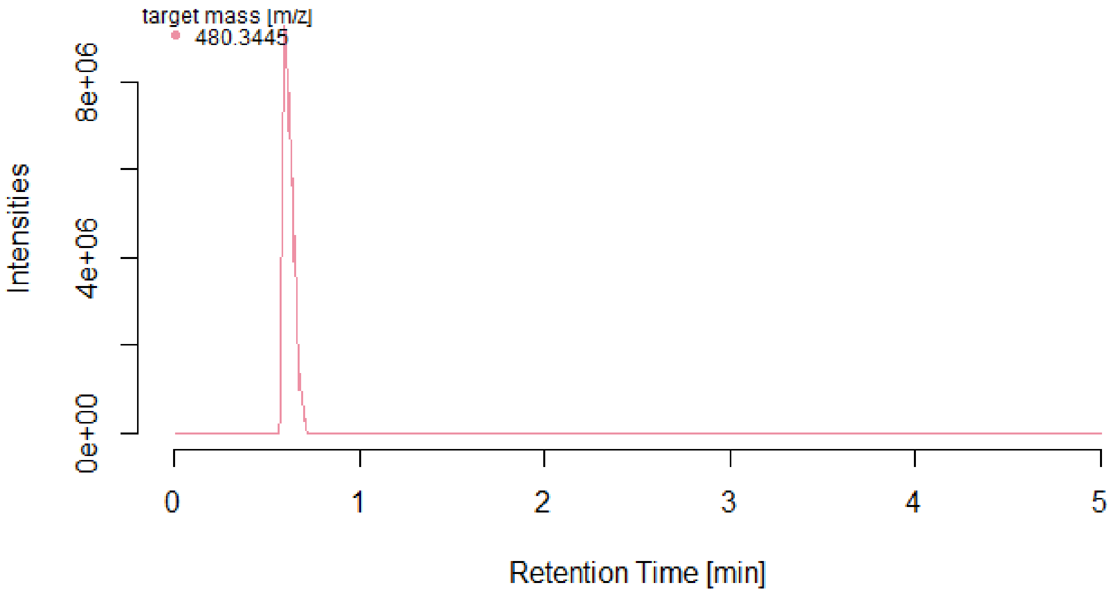

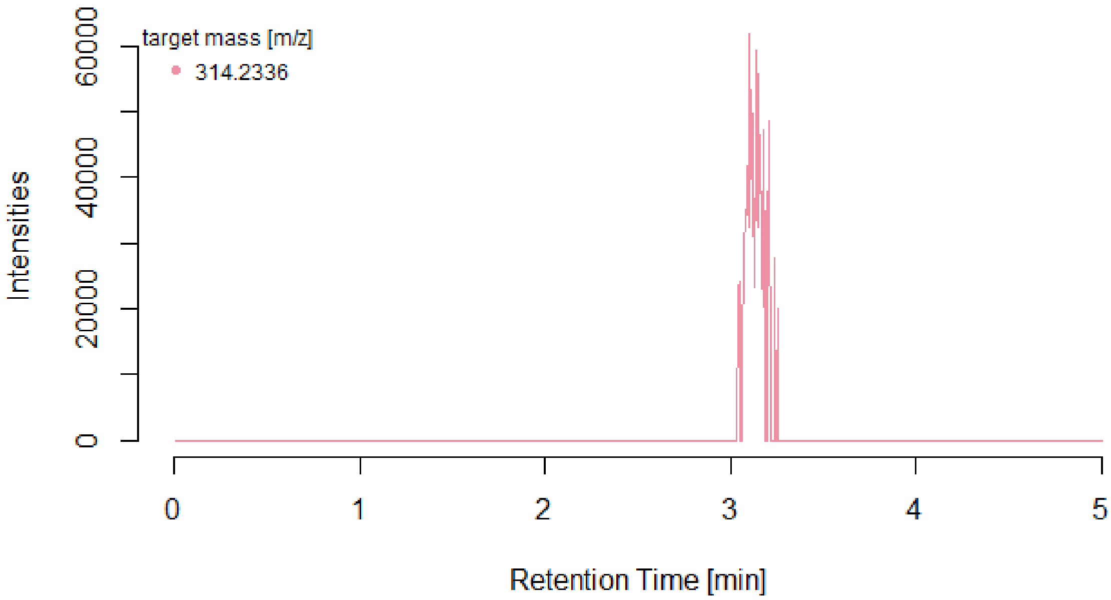

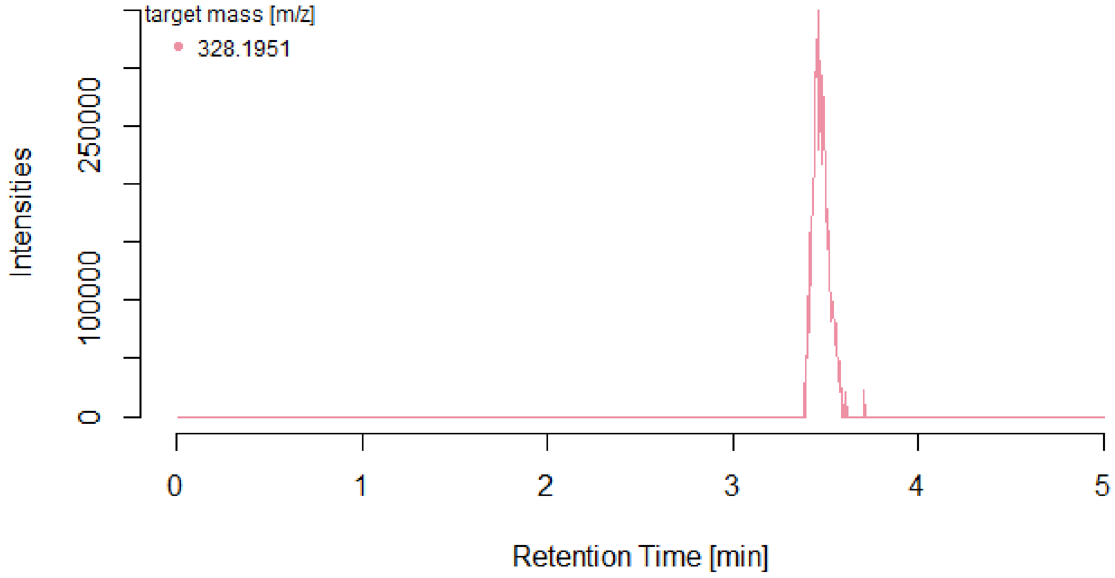
target mass [m/z]
328.1951
Intensities
0
100000
250000
0
1
2
3
4
5
Retention Time [min]

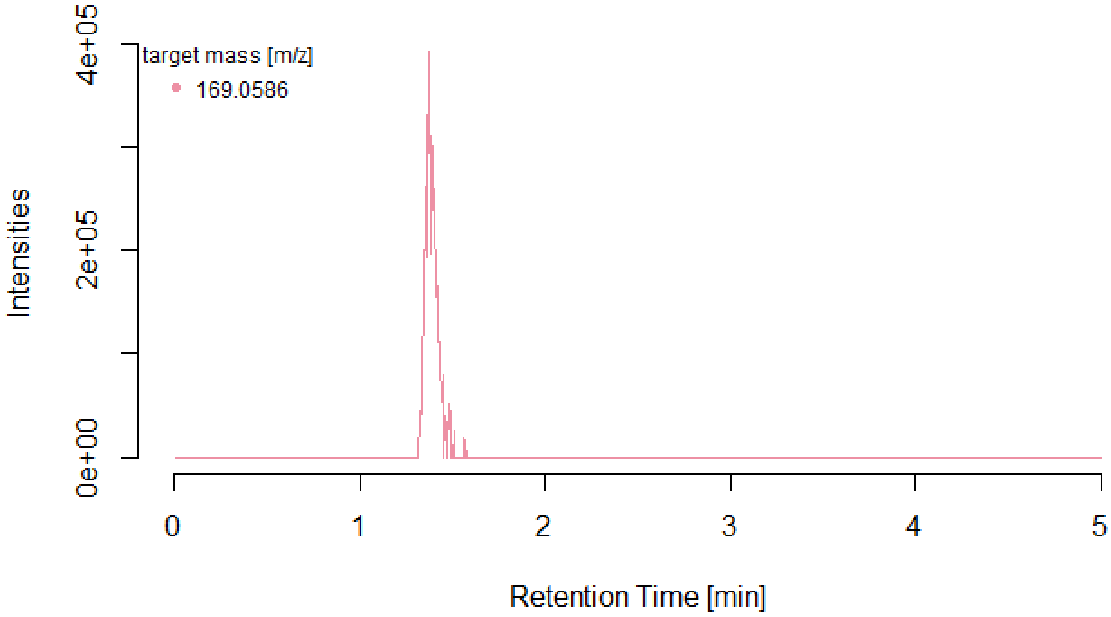

target mass [m/z]
169.0586
Intensities
0e+00
2e+05
4e+05
0
1
2
3
4
5
Retention Time [min]
