# Supplementary material for: Impact of Low-Level Ergot Alkaloids and Endophyte Presence in Tall Fescue Grass on the Metabolome and Microbiome of Fall-Grazing Steers
Source: Toxins (Basel). 2025 May 17;17(5):251. doi: 10.3390/toxins17050251 (PMC12115782; doi:10.3390/toxins17050251)
Supplement: Supplementary file 1 [file toxins-17-00251-s001.zip › toxins-3582876_Supplementary File 2.pdf]

Impact of low-level ergot alkaloids and endophyte presence in tall fescue grass on the metabolome and microbiome of fall grazing steers

Ignacio M. Llada, Jeferson M. Lourenco, M. Mikayla Dycus, Jessica M. Carpente, Garret Suen, Nicholas S. Hill, Nikolay M. Filipov

Table S1. Shannon diversity index, Faith's phylogenetic diversity index, species evenness, and number of observed features detected in the ruminal content, and feces of Angus steers grazing toxic endophyte-infected (E+; n=6), non-toxic endophyte-infected (NT; n=6), and endophyte-free tall fescue E- (E-; n=6), as well as in the fescue plants themselves (3 paddocks/treatment).

**Index**	**Rumen solid**				
	**E-**	**NT**	**E+**	**SEM**	**p-value**
	8.045	8.026	7.787	0.107	0.22
	38.587	39.237	38.331	1.011	0.81
	0.876	0.868	0.851	0.009	0.11
	599.667	626.038	579.967	30.356	0.59
	**Rumen Fluid**				
**Shannon index**	7.533	6.971	7.306	0.616	0.23
**Faith's PD**	61.033	59.101	61.934	1.458	0.41
**Evenness**	0.788	0.744	0.767	0.018	0.26
**Obs. Features**	778	685.636	752.6	43.427	0.34
	**Feces**				
	7.369	7.508	7.496	0.106	0.6
	30.055	30.05	31.192	0.897	0.56
	0.846	0.861	0.85	0.007	0.42
	426.5	426.23	471.067	28.632	0.46
	**Tall fescue plant**				
	2.07	2.074	2.039	0.404	0.99
	3.303	3.142	3.236	0.44	0.96
	0.436	0.426	0.46	0.07	0.93
	26.533	28.133	23.333	5.23	0.81

Data are presented as mean ± SEM, with significance determined at α=0.05 for all comparisons.

Table S2. The relative abundance (%) of the main families, genera, and Archaea in the rumen fluid, solid, and feces that significantly changed in steers grazing on tall fescue infected with toxic endophyte (E+; n=6), compared with steers grazing non-toxic endophyte (NT; n=6) and endophyte-free (E-; n=6), over the 28-day study period.

**Taxa (Family and genus level)**	**E-**	**E+**	**NT**	**p-value**
		**Rumen fluid**		
Prevotellacea	25.6^{a}	30.9^{b}	25.0^{a}	0.05
Christensenellaceae_R-7_group	6.0^{a}	4.7^{b}	6.3^{a}	0.03
Butyrivibrio	2.5^{a}	1.4^{b}	1.8^{ab}	>0.01
Solibacillus	0.4^{a}	1.3^{b}	0.3^{a}	0.05
Methanobrevibacter	88.1^{a}	78.8^{b}	85.7^{ab}	0.03
Methanomethylophilaceae_uncultured	9.0^{a}	17.2^{b}	10.8^{ab}	0.02
		**Rumen solid**		
Eubacterium_coprostanoligenes_group	0.6^{a}	0.4^{b}	0.6^{a}	>0.01
		**Feces**		
Rikenellaceae	9.4^{a}	11.8^{b}	9.7^{a}	>0.01
Clostridiaceae	0.7^{a}	0.3^{b}	0.7^{a}	0.05
Rikenellaceae_RC9_gut_group	6.0^{a}	8.0^{b}	6.2^{a}	0.01
Clostridioides	1.1^{a}	0.7^{b}	1.0^{a}	0.01
*Clostridium_sensu_stricto_*	0.7^{a}	0.3^{b}	0.7^{a}	0.05

Mean values with a superscript in common do not differ with a level of α=0.05 over all comparisons

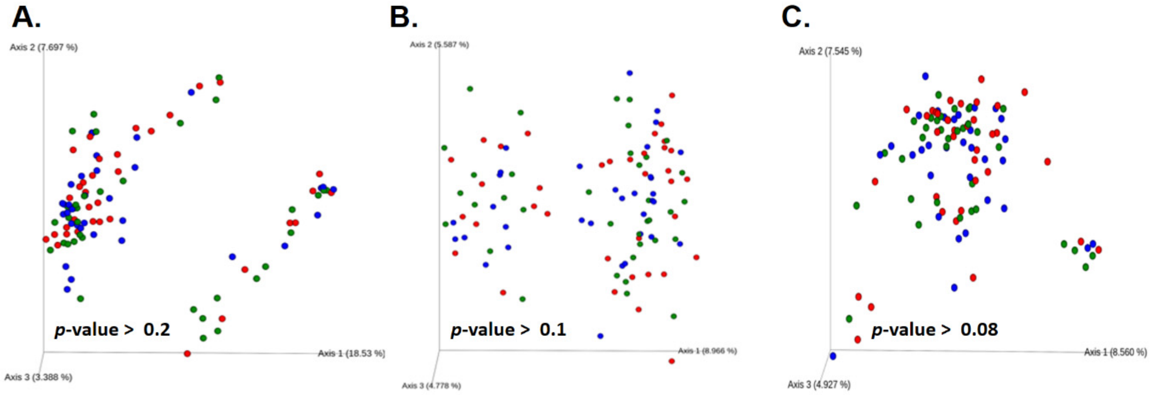

Figure S4: Principal coordinate analysis plot of beta diversity (unweighted UniFrac) of bacterial population in the ruminal fluid (A), solid (B), and fecal (C) samples of steers (n = 18) grazing toxic endophyte-infected tall fescue (E+; n=6), non-toxic endophyte-infected tall fescue (NT; n=6), and endophyte-free tall fescue E- (E-; n=6). Significance determined at α=0.05 for all comparisons.

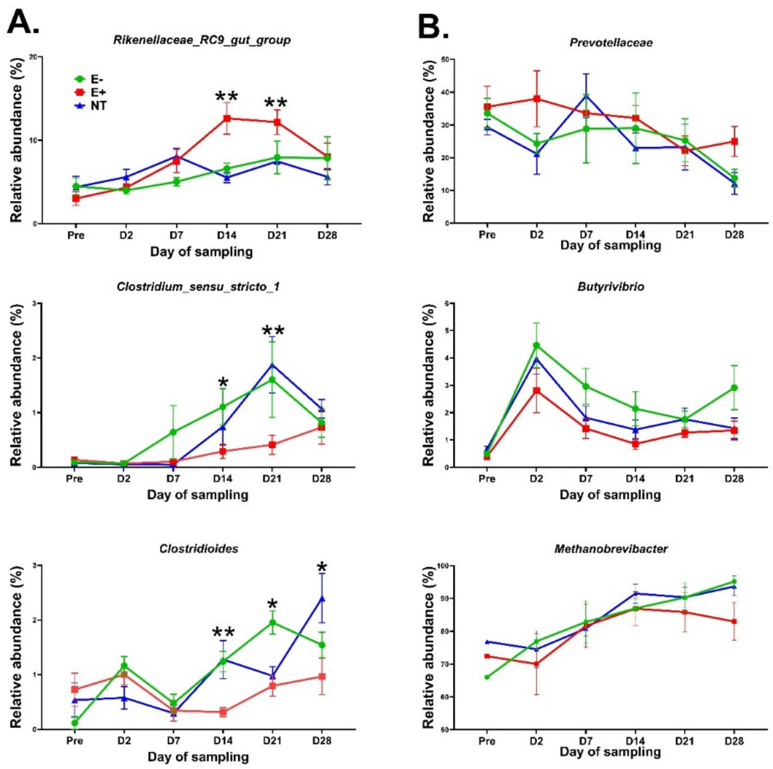

Figure S5: Relative abundance by day of sampling for selected bacterial and archaeal taxa that showed an overall effect in feces (column A) and rumen fluid (column B). (*) indicates a significant difference ($P \leq 0.05$) between the E+ group and one of the other groups, while (**) indicates a significant difference ($P \leq 0.05$) between the E+ group and both other groups. Data are presented as percentages (%) and expressed as mean ± SEM.
